# Topics in Antivax and Provax Discourse: Yearlong Synoptic Study of COVID-19 Vaccine Tweets

**DOI:** 10.2196/45069

**Published:** 2023-08-08

**Authors:** Zainab Zaidi, Mengbin Ye, Fergus Samon, Abdisalan Jama, Binduja Gopalakrishnan, Chenhao Gu, Shanika Karunasekera, Jamie Evans, Yoshihisa Kashima

**Affiliations:** 1 Melbourne School of Psychological Sciences University of Melbourne Parkville Australia; 2 Centre for Optimisation and Decision Science Curtin University Perth Australia

**Keywords:** COVID-19 vaccine, vaccine hesitancy, antivax, stance detection, topic modeling, misinformation, disinformation

## Abstract

**Background:**

Developing an understanding of the public discourse on COVID-19 vaccination on social media is important not only for addressing the ongoing COVID-19 pandemic but also for future pathogen outbreaks. There are various research efforts in this domain, although, a need still exists for a comprehensive topic-wise analysis of tweets in favor of and against COVID-19 vaccines.

**Objective:**

This study characterizes the discussion points in favor of and against COVID-19 vaccines posted on Twitter during the first year of the pandemic. The aim of this study was primarily to contrast the views expressed by both camps, their respective activity patterns, and their correlation with vaccine-related events. A further aim was to gauge the genuineness of the concerns expressed in antivax tweets.

**Methods:**

We examined a Twitter data set containing 75 million English tweets discussing the COVID-19 vaccination from March 2020 to March 2021. We trained a stance detection algorithm using natural language processing techniques to classify tweets as *antivax* or *provax* and examined the main topics of discourse using topic modeling techniques.

**Results:**

Provax tweets (37 million) far outnumbered antivax tweets (10 million) and focused mostly on vaccine development, whereas antivax tweets covered a wide range of topics, including opposition to vaccine mandate and concerns about safety. Although some antivax tweets included genuine concerns, there was a large amount of falsehood. Both stances discussed many of the same topics from opposite viewpoints. Memes and jokes were among the most retweeted messages. Most tweets from both stances (9,007,481/10,566,679, 85.24% antivax and 24,463,708/37,044,507, 66.03% provax tweets) came from *dual-stance* users who posted both provax and antivax tweets during the observation period.

**Conclusions:**

This study is a comprehensive account of COVID-19 vaccine discourse in the English language on Twitter from March 2020 to March 2021. The broad range of discussion points covered almost the entire conversation, and their temporal dynamics revealed a significant correlation with COVID-19 vaccine–related events. We did not find any evidence of polarization and prevalence of antivax discourse over Twitter. However, targeted countering of falsehoods is important because only a small fraction of antivax discourse touched on a genuine issue. Future research should examine the role of memes and humor in driving web-based social media activity.

## Introduction

### Background

Discourse on vaccination began early during the COVID-19 pandemic and has since received sustained attention in mainstream media and social media and among the general public around the world. Although the widespread uptake of COVID-19 vaccines in many countries has enabled them to shift toward *living with COVID*, the emergence of new variants and the inevitable waning of immunity mean that vaccination remains central to the world’s capability of coping with future infection waves [1]. At this juncture, understanding the public vaccination discourse and its dynamics is critically important for governments, policy makers, and scientists to maintain and increase the trust and uptake of vaccines in the future. By conducting a comprehensive study of English-language Twitter activities, we attempted to demystify web-based vaccination discourse and provide a better understanding of its content and dynamics.

Existing research on COVID-19 vaccine discourse has focused on antivaccination misinformation, examining its spread over social media and its influence on the vaccination debate [2-7]. Mainstream media have frequently reported on and fact checked misinformation and falsehoods related to COVID-19 vaccines [8-15]. Over the course of the last 2 years, a perception has emerged that discussion around COVID-19, especially about vaccination, is highly polarized. In this view, anti- and provaccination discourses run in parallel without interacting with each other; each coalescing around shared narratives while ignoring the information and arguments that challenge them [16,17].

Contrary to this popular perception, we are yet to have a clear picture of the content and dynamics of public discourse on COVID-19 vaccines on social media. This is largely because of the absence of a reliable and scalable method for stance detection, namely, measuring a pro- or antistance on an issue expressed in social media messages. First, we do not know whether provax or antivax messages predominate the discourse on social media. On the one hand, manual coding [4,18] has found relatively more antivax than provax tweets, but this method is unscalable and limited to a few thousand randomly sampled tweets. On the other hand, scalable unsupervised learning methods, such as sentiment analysis and topic modeling, are not always reliable and can yield contradictory results. Although studies using sentiment analysis have reported prevalent positive sentiments about vaccines over Twitter [3,6,19,20], a topic modeling approach to stance prediction in the study by Yousefinaghani et al [6] classified relatively more tweets as antivax than provax.

Similarly, it is still unclear whether vaccination discourse is indeed polarized. Although human messaging, rather than bot activities, appears to shape web-based discourse [2], the most prolific of those human contributors do not appear to show a strongly polarized vaccination stance. Gori et al [4] found this to be the case based on manually annotated Italian tweets around the time of vaccination campaigns. Intriguingly, however, these users’ tweet contents were extremely polarized. The trend of sentiments further complicates the picture, with Greyling and Rossouw [21] reporting a global downward trend in positive sentiments toward vaccination over 6 months and [22] showing a general negative or neutral trend. Does this mean antivaccination discourse is gaining the upper hand?

Furthermore, if indeed the web-based discourse is responsible for changing sentiments, we still do not know what information and arguments affect vaccination stances. Previous studies have suggested that safety, mistrust of government and pharmaceutical companies, accessibility issues, conspiracies, and misinformation are key barriers to vaccine uptake [18,20,23]. Topic modeling of vaccine-related tweets [24] identified opinions on vaccination, vaccine progress, and instructions on receiving vaccines as the main topics. However, neither study examined vaccination stance and therefore could not examine which topic was associated with which stance. A few studies [5,25,26] have used the Bidirectional Encoder Representations from Transformers model for stance detection of COVID-19 vaccine–related tweets, trained with 1500 to 15,000 labeled tweets. Hayawi et al [5] demonstrated the efficacy of the model in stance detection; however, they did not examine the topics being discussed. Poddar et al [26] analyzed 15 million tweets and found 12 most important topics, and Jiang et al [25] focused on the association of vaccine stance and political polarization.

### Objectives

In this study, we focus on the following research questions (RQs):

- RQ1: What topics were discussed in both antivax and provax camps relevant to COVID-19 vaccines?
- RQ2: Is it possible to explain the significant peaks in the volumes of antivax and provax tweets in relation to significant COVID-19–related events?
- RQ3: Diving deeper into antivax tweets, can we assess the genuineness of the concerns and issues expressed there?

To gain a comprehensive understanding of the content and dynamics of social media discourse on COVID-19 vaccination, we examined 75 million English-language tweets related to COVID-19 vaccines. These tweets were extracted using vaccine-related keywords over a yearlong observational period from March 20, 2020, to March 23, 2021, from a publicly available data set of COVID-19 tweets collected by Lamsal [27]. We used natural language processing and OpenAI’s GPT transformer–based stance detection tool [28,29], trained on a subset of 46,176 manually labeled tweets, to classify antivax and provax tweets. Both classes were then analyzed to determine discussion topics using a composite strategy involving the Gibbs Sampling Dirichlet Multinomial Mixture (GS-DMM) topic modeling tool [19] and a manual search to compile a comprehensive list of topics and relevant keywords and phrases. The details of this method are presented in the next section.

An interesting finding of the initial data analysis is a significantly large set of user IDs, 1,893,232 out of 8,637,015 unique user IDs (for antivax and provax tweets), who have posted both antivax and provax tweets, which leads to our fourth and final RQ:

- RQ4: Is this dual-stance cohort an artifact of stance detection noise or are there many users who were expressing opposing stances? If the latter is true, what are they talking about and how does their content contrast with the tweets of the users posting only antivax or only provax tweets?

This cohort is discussed in detail in the *Results* section.

## Methods

### Data Set

This study used the publicly available data set collected and maintained by Lamsal [27] and contained tweet IDs for global English-language tweets featuring COVID-19–related keywords. A complete list of keywords can be found in the study by Lamsal [27]. We started hydrating Lamsal’s data set in February 2021, which involved retrieving the tweets and associated attributes using the tweet ID. We observed that 25.77% (602,790/2,338,713) to 39.78% (330,666/831,327) of tweets could not be retrieved from March to April 2020. This hydration loss gradually decreased to 10.86% (288,563/2,656,607) to 12.6% (283,383/2,249,082) from February to March 2021. To extract the vaccine-related tweets, we further filtered the data set with the keywords *vaccine*, *vaccination*, *vax*, *vac*, *jab*, and *shot*.

### Stance Detection

Our study used a stance detection tool created from OpenAI’s GPT transformer model [29] to classify tweets into 3 categories (ie, favor, against, and none) for the topic *vaccine hesitancy*. Transformer has proven to be a very powerful language modeling tool mainly because of the self-attention mechanism, which can capture deeper context and long-range language characteristics better than long short-term memory–based approaches [28]. The transformer language model is already pretrained in an unsupervised manner but requires supervised fine-tuning for target tasks, such as stance detection. The details of the stance classification tool and its performance can be accessed from [28]; here, we discussed the details of its application to COVID-19 vaccine tweets.

To make this yearlong study possible with reasonable confidence, we had 46,176 tweets to label. As the conversation was changing throughout the year, it was critical to have a relevant labeled set of tweets to fine-tune the GPT model, which can then be used to detect stances for the tweets of that specific period. The classification accuracy, measured in terms of the composite *F*-score for favor and against classes [28], depends critically on the labeled set used to fine-tune the GPT model. After various trials, we ended up sampling approximately 100 random tweets from each day, which gave us reasonable *F* scores of ≥0.6 for 20 test sets we picked and labeled from the yearlong data set [27]. Test sets comprised 250 to 1000 randomly selected tweets from the data collected on the 20th, 40th, 60th,..., 360th day. In total, 41,911 labeled tweets were used for training, and 20 test sets contained 4265 labeled tweets. As described in a previous study [28], 20% of the training samples were used for validation. The batch size was maintained at 2 because of Graphics Processing Unit memory limitations, and the training loop was executed for 3 epochs, as in [28]. The composite *F* scores for the 20 test sets were in the range of 0.67 to 0.87. The average precision was 0.74 (SD 0.17) for antivax class and 0.84 (SD 0.08) for provax class. The mean recall was 0.71 (SD 0.11) for antivax class and 0.88 (SD 0.09) for provax class, which resulted in mean *F*-score of 0.71 (SD 0.11) for antivax class and 0.86 (SD 0.07) for provax class.

Considering the enormous task load, we devised an annotation strategy comprising 2 phases. In the first phase, a primary annotator provided 1 set of labels for all 46,176 tweets. The second phase focused on estimating the bias error in the annotation by the primary annotator from the first phase. Extra annotators provided at least 2 additional sets of independent labels for 3000 tweets from March to April 2020 and 3000 tweets from September to October 2020, and a majority rule was applied to obtain the final label. There was 93% similarity between the final labels and primary annotations for March to April 2020 and 89% for September to October 2020. We remarked that the period from September to October 2020 was exceptionally complex for annotation because of an ongoing contentious debate between Democrat and Republican voters in the United States, and leaders from both sides were called antivax. We labeled all such political tweets with the stance *none*, noting that it was not always clear if the main focus of the tweet was vaccines or politics.

The stance prediction results when the GPT model was fine-tuned using both sets of labels, that is, final labels and primary annotation only, were 91% similar for the March to April 2020 period and 84.1% similar for the September to October 2020 period. Both sets of labels resulted in similar predictions. Thus, we argue that bias error is marginal in this study and can be safely ignored. Moreover, any misclassification of tweets due to personal bias can be handled, along with the prediction error of the GPT model, when each class is thoroughly analyzed for specific discussion points through subsequent topic modeling.

### Topic Modeling

Latent Dirichlet allocation (LDA) topic modeling relies on the co-occurrence of words in a document, and a major issue with tweets, which comes under Short Text Topic Modelling, is that it is limited to a maximum of 280 characters [30]. We tested several topic modeling tools, such as LDA [31], GS-DMM [19], Bertopic [32], and Top2vec [33], and found that GS-DMM, an LDA-based topic modeling tool optimized for Short Text Topic Modelling, as it clusters similar short text documents together using the movie group process [19], was most suitable in finding a relatively distinct and meaningful cluster of words. However, GS-DMM alone was not sufficient for the topic classification tasks because, as it turns, the core topics are highly intertwined with one another and use similar words.

We then decided to use GS-DMM for keyword collection instead and added another phase in our strategy, where these keywords were manually searched for in the tweet files and key phrases were selected for final topic classification. For example, the word *force* appearing in an output word cluster of GS-DMM could be referring to the topic of *vaccine mandate*. However, if we use *force* as a keyword for the topic, it will also classify many irrelevant tweets, such as those talking about the good work done by the United States coronavirus task force, in addition to relevant tweets, such as those stating that *people cannot be forced to get the vaccine*. The second phase in our topic modeling strategy is linked to extending keywords into key phrases. Here instead of *force*, *cannot be forced* is a better choice for topic classification for *vaccine mandate*. The key phrases considerably lowered the misclassification rate. We sorted unique tweets for each topic according to the number of retweets found in the data set and manually checked 50 highly retweeted tweets for each topic to calculate the error rate. More than half of the topics have 0 errors, and only 7 topics have an error rate higher than 10%. The topic of *RNA altering DNA* had the highest error rate of 25%, *side effects* had 19% error, and 16.8% error was seen in the topic of *microchip*, but these errors appear to be mostly because of the stance misclassification of satirical tweets. The topic *Bill Gates* had an error rate of 14.7% for the antivax class, which is because of the misclassification of tweets that discussed legislative bills and or Bill Clinton.

### Network Graph

The 2 main tools used to build a retweet-directed network were NetworkX and Gephi. In a retweet network, users were represented by nodes, and retweet relationships were represented by edges. Edges were directed from the original author to the retweeting user. The retweeted users and the original tweeted users in the database were extracted to construct an edge table. NetworkX was then used to convert the edge table into a network file. The Gephi software was used to visualize the network file.

### Ethics Approval

This research analyzed publicly available tweets and user data and reported the results in a responsible and ethical manner. This study was reviewed and approved by the ethics committee of the Office of Research Ethics and Integrity of the University of Melbourne (Application Ref 2023-26494-37214). The consent waiver was granted because the use of public tweets for aggregate analysis falls within the expected and publicly known use of Twitter content. The reidentifiability of individual users through verbatim examples was prevented by paraphrasing content where needed. Data sharing was aligned with Twitter policies.

## Results

### Overview

**Figure 1:**
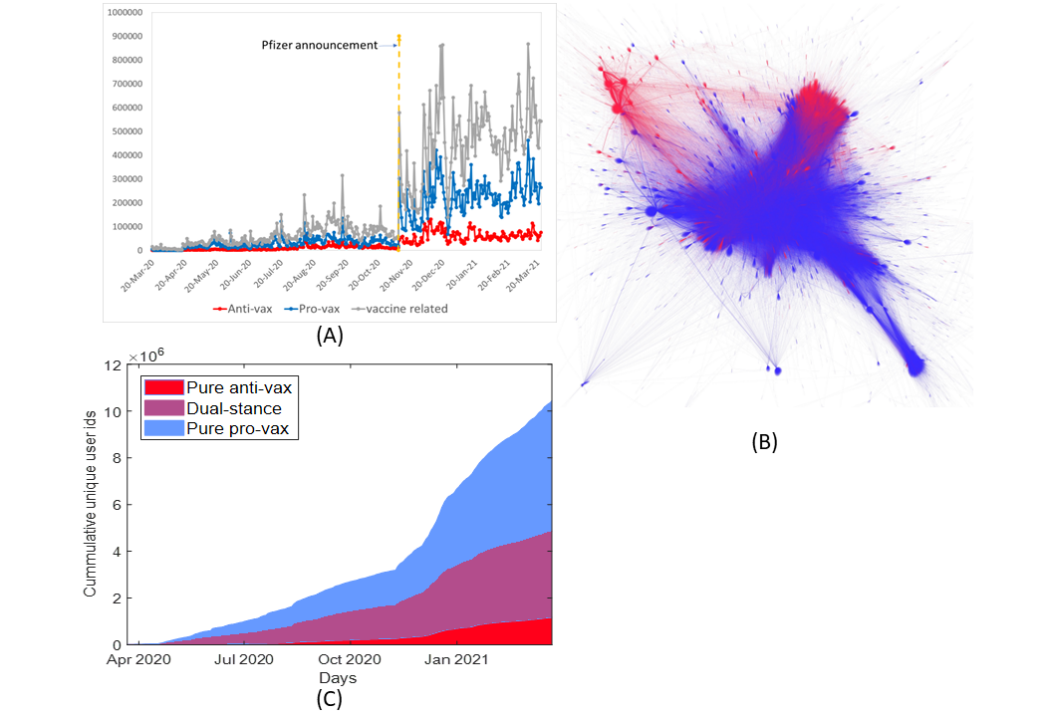
One year of COVID-19 vaccine tweets, from March 20, 2020, to March 23, 2021, are analyzed in this study. The tweets are classified into antivax and provax tweets through natural language processing–based stance detection. A 10-fold increase in vaccine-related tweets is observed after November 9, 2020, when Pfizer announced the results of their preliminary analysis [32]. (A) Time series of total COVID-19 vaccine–related tweets from March 2020 to March 2021 and its breakdown into antivax and provax tweets. (B) The network of users on Twitter, with directed links to the retweeting users from the users who posted the original tweet. Blue and red links represent a provax and antivax retweet, respectively, whereas violet links represent both provax and antivax tweets originated by one user and retweeted by the other user. Retweets classified as neutral or irrelevant are not included. (C) The number of unique user IDs throughout the year for pure antivax, dual-stance, and pure provax groups steadily increased, with a higher rate of increase in the postvaccine period.

Our stance detection algorithm showed that provax tweets clearly outnumbered antivax tweets throughout the observation period (Figure 1A): 37*,*047*,*378 provax; 10*,*567*,*955 antivax; and 28*,*322*,*526 neutral or irrelevant tweets. Moreover, of the 8,637,015 unique user IDs (for antivax and provax tweets), 5*,*571,946 (64.5%) tweeted only provax messages, whereas 1*,*171,837 (13.6%) tweeted only antivax messages. Most intriguingly, 1*,*893,232 (21.9%) of 5*,*571,946 users sent out both provax and antivax tweets (Figure 1C), whose presence was hinted at by Gori et al [4]. Figure 1B shows the communication network, in which links represent retweets and nodes represent users (see the *Methods* section). Although there appeared to be several like-minded clusters, they were far from isolated. There seems to be a small (red) antivax cluster. On the basis of the data, two broad observations were made in this study: (1) provax discourse is predominant in the English-language Twittersphere, with >85% of the users posting a provax tweet; and (2) we found limited evidence of polarized discourse. A majority of those who sent an antivax tweet also sent out a provax tweet (1*,*893,232/3,065,069, 61.8%). This cohort is discussed in more detail in this section after the presentation of the topics discussed in the vaccine debate.

Using topic modeling, we found that although a single topic—*vaccine development*—dominates provax tweets, antivax tweets mention more diverse topics, including *vaccine mandates*, *vaccine side effects*, and *masks and lockdowns*. Some topics commonly appeared in both antivax and provax tweets (eg, *masks and lockdowns* and *Pfizer and Moderna vaccines*) with opposing perspectives. Memes and humorous tweets (classified as the topics *jokes* and *jokes (side effects)*) were among the most retweeted messages for both the antivax and provax tweet sets, with 7 of the 10 most retweeted antivax tweets involving memes or jokes. In fact, they dominated the tweets of those who tweeted only provax or antivax messages. Most of the antivax tweets contained falsehoods (eg, *COVID-19 is a hoax* and *COVID-19 is a secret plan to limit people’s rights*), and only 10% to 15% expressed genuine concerns (eg, mandatory vaccination and blood clots following the AstraZeneca [AZ] vaccine).

### The Vaccine Debate

Figure 1A presents the yearlong time series of the volume of tweets classified as provax (blue) and antivax (red) and the total number of tweets (gray). The first notable feature is a 10-fold increase in vaccine-related tweets from November 9, 2020, which coincides with Pfizer announcing preliminary efficacy results for their COVID-19 vaccine [34]. We used this announcement date to divide the year into 2 significantly different periods, namely, the *prevaccine* and *postvaccine* periods. The second significant aspect is that the number of provax tweets is over 3 times that of antivax tweets, noting that provax tweets contain tweets from governments, pharmaceutical companies, and media agencies, in addition to regular users.

Figure 1C shows the growth of unique user IDs associated with pure antivax (red), dual-stance (violet), and pure provax (blue) tweets over the year. Both classes of antivax and provax also exhibit almost identical user growth rates—lower and higher in the prevaccine and postvaccine periods, respectively. However, provax tweets have a higher growth rate than antivax tweets.

**Figure 2:**
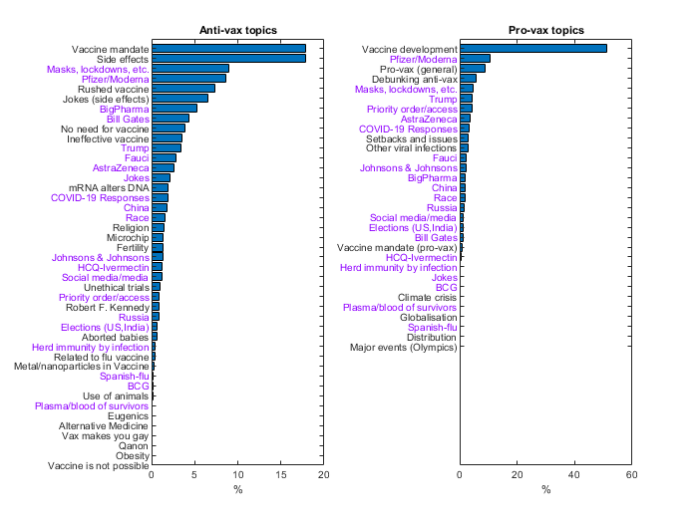
Using topic modeling techniques and manual search, 43 topics are found for antivax tweets and 31 topics are found for provax tweets, with 21 common topics (shown in violet color). The topics that are identified in the antivax and provax tweets are ranked according to the fraction of tweets containing the given topic. BCG: Bacillus Calmette–Guérin vaccine; HCQ: Hydroxychloroquine medicine.

Using topic modeling, we identified 53 distinct discussion topics, with 31 and 43 topics for provax and antivax tweets, respectively. A total of 21 topics were common across both the classes. Note that the topics were not mutually exclusive, and one tweet can contain multiple topics, whereas 21.15% (7,836,539/37,044,507) to 25.49% (2,693,640/10,566,679) of both provax and antivax tweets were not classified into any topic. Figure 2 shows the percentages of tweets for the identified topics relative to the total number of provax and antivax tweets. One striking difference is the diversity of topics covered by antivax tweets. Although *vaccine development* (18,989,506/37,044,507, 51.26%) dominates provax tweets, antivax tweets cannot be summarized with a few topics. The top antivax theme (3,119,651/10,566,679, 29.52%) is *vaccine safety*, a combination of *side effects*, *rushed vaccine*, and *jokes (side effects)*, followed by *vaccine mandate* (1,887,323/10,566,679, 17.86%), and all other topics with a <10% share. Note that we separated the humorous tweets into 2 groups, with jokes regarding side effects in *jokes (side effects)* and the rest in *jokes*, mainly to quantify safety concerns.

Next, we reported the key topics (details about less significant topics are in Multimedia Appendix 1 [8-10,28,35-90]) and their dynamics over time by plotting daily tweets and linking their significant peaks to COVID-19–related current events.

### The Antivax Tweets

#### Timeline in Relation to Key Events

**Figure 3:**
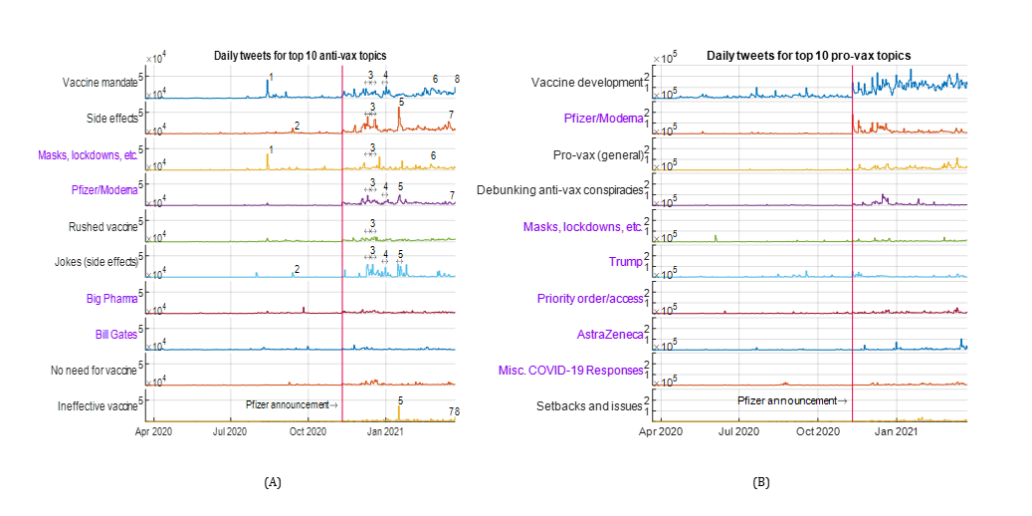
Vaccine mandate and the broad theme of safety, which includes side effects, rushed vaccine, and jokes (side effects), are the most significant topics in antivax discussion, whereas the provax tweets are dominated by updates around vaccine development. November 9, 2020, marks the start of the postvaccine period, with Pfizer announcing the results of their trials. (A) The top 10 topics in antivax class and (B) the top 10 topics in provax class.

We started with antivax tweets. Despite their relative infrequency, antivax tweets appeared to drive many significant vaccine discourse dynamics. The peaks numbered in Figure 3A of the top 10 antivax topics appear to be linked to COVID-19–related current events, as follows:

1. August 12, 2020—A peak in the topic *vaccine mandate* was observed, which is coincidently 1 day after the announcement of clinical outcomes for the Russian vaccine Sputnik. The tweets were not referring to Sputnik exclusively, but the increased likelihood of a vaccine in the near future might have triggered the mandate debate. This is the most significant peak in the prevaccine period, and the timing is interesting, even if it is not directly linked to the Sputnik announcement.
2. September 11, 2020—AZ’s phase 3 trials were paused on September 8, 2020, after 1 volunteer developed an unknown reaction [91]. Peaks were observed in the topics of *side effects* and *jokes (side effects)*.
3. December 7 to 20, 2020—Multiple peaks are observed in the topics of *side effects*, *Pfizer and Moderna*, *jokes (side effects)*, *rushed vaccine*, *vaccine mandate*, and *masks and lockdowns*, throughout the 2 weeks of December 2020, coinciding with the Pfizer-BioNTech and Moderna vaccines gaining various approvals in the United Kingdom and from the US Food and Drug Administration (FDA) [92-96], and the launch of UK’s public vaccination program on December 8, 2020.
4. December 28, 2020, to January 3, 2021—Several countries closed their borders to the United Kingdom in response to the Alpha variant of COVID-19 on December 21, 2020 [97], whereas the United Kingdom government changed the gap between Pfizer doses and approved the AZ vaccine on December 30, 2020 [98,99]. We observed higher tweet activity for *vaccine mandate*, *Pfizer and Moderna*, and *jokes (side effects)* on multiple days, including December 31, 2020.
5. January 15 to 17, 2021—A viral meme about zombie apocalypse because of failed vaccine in the movie I am Legend and its relevance to the current pandemic drove peaks in the *side effects, jokes (side effects)*, *Pfizer and Moderna*, and *ineffective vaccine* topics on January 15, 2021. From January 16 to 17, 2021, the most significant news shared under the jokes (side effects) and Pfizer and Moderna topics was about 23 deaths in Norway after vaccination [100]. The incident is discussed in detail in the sequel.
6. February 22 to 26, 2021—United Kingdom’s review of vaccine passports on February 23, 2020 [101] may be behind the spikes in *vaccine mandate* on February 24 and 26, 2021. On February 21, 2021, Dr Fauci stated that Americans may need to wear masks in 2022 [102], which might be linked to a peak in the plot of *masks, lockdowns, etc* the next day.
7. March 11 to 17, 2021—On March 11, 2021, the use of the AZ vaccine was suspended across many countries because of fears of blood clots [103] causing peaks in *side effects* on March 15, 2021, and *Pfizer and Moderna* on March 16, 2021. Coincidentally, the third COVID-19 wave was sweeping across Europe [104], and there were multiple spikes in the *ineffective vaccine* and *vaccine mandate* topics.

Although it is challenging to establish a causal link between a possible triggering current event and the topics discussed in the tweet data set, many of the antivax tweets appear to be spontaneous responses to key COVID-19 vaccine events. This suggests that it may be challenging to develop counternarratives in a timely manner and deploy them to *prebunk* vaccine-related misinformation [104]. Moreover, memes and funny tweets seem to play a significant role in the progression of antivax narratives, which we will return to in detail later. Next, we discuss major antivax topics in detail.

#### Safety (Side Effects, Rushed Vaccine, and Jokes [Side Effects])

Under the theme of *safety*, we group 3 topics, *side effects*, *rushed vaccine*, and *jokes (side effects)*, accounting for 29.52% (3,119,651/10,566,679) of the antivax tweets. Of these, *side effects* accounted for the largest number of tweets, discussing a broad range of illnesses because of receiving a vaccine dose, ranging from allergic reactions to death. Although some conspiracies found their way into the conversation, such as vaccines causing infertility, some tweets included elements of true adverse reactions from vaccine trials. Nonetheless, many were exaggerations of facts, quoting facts out of context, and even falsehoods regarding incidents that had never occurred (see Multimedia Appendix 1 for details). For example, media coverage on the 23 elderly people (ie, in an aged-care home) who died in Norway after taking the Pfizer vaccine in January 2021 [100,105] was shared in 12,249 tweets and caused a peak in *Pfizer and Moderna* and *jokes (side effects)* topics in Figure 3A on January 16 to 17, 2021. An investigation by Norwegian Medicines Agency, reported by Torjesen [100], stated “common adverse reactions to mRNA vaccines may have contributed to worsening of their underlying diseases and a fatal outcome in some frail patients”—this article was subsequently shared by 3323 tweets that suggested vaccines “may have led” to deaths. Shortly thereafter, our data set showed thousands of tweets discussing an increase in the number of deaths in Norway.

The plot of *rushed vaccine* was consistent in the postvaccine period. The peak on August 12, 2022, was possibly triggered by the announcement of the Sputnik vaccine. The tweets were typically either falsehoods or cherry-picked information taken out of context, highlighting the extremely rapid development and approval processes, in contrast to previous vaccine development efforts. These doubts can be related to mistrust over authorities and pharmaceutical companies, as reported by Lanyi et al [20] and Küçükali et al [23]. An example is the discussion about hydroxychloroquine (see Multimedia Appendix 1 for details), which was banned as a possible COVID-19 treatment after multiple scientific studies [35]; however, the antivax tweets suggested that hydroxychloroquine was banned to give emergency approval to COVID-19 vaccines and give pharmaceutical companies greater profits. Close to the US elections, the political war-of-words also kicked in, where anti-Trump users said they were not ready to trust a vaccine announced by Trump, calling it rushed, and pro-Trump users called them antivax. Opposing political blocs were also seen pointing fingers at each other’s measures to fast-track the vaccine. The major concerns discussed under *rushed vaccine* were about an experimental vaccine, which was made in less than a year, and the fact that long-term side effects are not known. The typical process of multiphase (at least 3 phases preregistration) clinical trials for new vaccines may take ≥10 years [106]. However, there is an obvious need to fast-track the COVID-19 vaccine process. The Sputnik vaccine was announced on August 11, 2020, after phases 1 and 2 of the trials, and phase 3 trials commenced postregistration [107] and reported >90% efficacy. The Pfizer vaccine was announced on November 9, 2020, after the first interim data analysis of phase 3 trials, and it received the FDA’s Emergency Use Authorization on December 11, 2020, after the conclusion of the phase 3 trials.

*Jokes (side effects)* are discussed later under *Memes* section because they appear to have somewhat different dynamics.

#### Vaccine Mandate

The single most significant topic discussed in antivax tweets is *vaccine mandate*. The expressed opinions ranged from mandates infringing on basic human freedom (if vaccination was required to travel, shop, dine, and be employed) to the use of mandates for control and profit. For instance, some tweets suggested that the coronavirus was purposely released or that the media was exaggerating the pandemic, to create business opportunities for pharmaceutical companies, or to limit people’s rights as a secret plan to create an Orwellian totalitarian dystopia. Moreover, virus control measures, such as masks and lockdowns, are described as instruments for establishing control and coercing people into accepting a vaccine.

On August 21, 2020, World Health Organization’s director said in a media briefing: “we cannot go back to the way things were” [108]. Several tweets referred to this statement to suggest that the COVID-19 pandemic is about changing society and not about a virus. These tweets contributed to the *vaccine mandate*’s peak on September 3, 2020. Protests and vaccine refusals from America’s front-line doctors [109], a group of physicians against vaccines, and other medical professionals are also discussed in many tweets [23]. Later, we attempt to separate conspiracies and falsehoods from the discussion of genuine concerns to understand the composition of the antivax discourse.

We also found discussions about historical debates around parental consent within the context of childhood vaccinations, the slogan of *my body my choice* typically associated with abortion rights movements [110], efficacy issues of influenza vaccines, and comparisons between voter IDs and vaccine certificates. Some tweets argued that taking a fast-tracked vaccine without fully understanding the long-term effects can only be an individual’s choice. Other tweets suggested that making vaccines mandatory is not a solution, and we should just learn to live with the virus.

#### Vaccine Efficacy (No Need for Vaccine and Ineffective Vaccine)

We grouped *no need for vaccine* and *ineffective vaccine* under the theme of vaccine efficacy. Surprisingly, this theme did not receive major traction during our study period, although vaccine efficacy may have become a more significant point of discussion in the second year of the pandemic, with the emergence of the Delta and Omicron variants of COVID-19.

The tweets on *ineffective vaccine* mainly highlighted and propagated the doubts raised by authorities, who at the time were uncertain if vaccination alone could resolve the pandemic. On December 28, 2020, a World Health Organization expert said, “At the moment, I don’t believe we have the evidence on any of the vaccines to be confident that it’s going to prevent people from actually getting the infection” [108]. Many tweets twisted this statement to “there is no evidence that vaccine will prevent infection.” These tweets have basically exploited the uncertainties associated with vaccine efficacy and have taken the statements out of context to align with the antivax narrative. The only significant peak in *ineffective vaccine* occurred on January 15, 2021, similar to the tallest peak in the *side effects* topic and is due to a meme about a zombie apocalypse resulting from a failed vaccine in the movie *I am Legend*.

On *no need for vaccine*, some tweets focused on the advice about using masks after vaccination, inferring (wrongly) that vaccines are pointless if masks are still needed. Moreover, the discussions on this topic mostly compared COVID-19 with influenza, and the common cold and claimed COVID-19 had a survival rate >99.7%. Multiple peaks in *no need for vaccine* topic occurred from December 7 to 20, 2020, when the vaccines from Pfizer and Moderna were approved by the FDA [93,96] (see the above discussions about December 7 to 20, 2020).

### The Common Topics

The provax and antivax tweets discussed common topics from opposing viewpoints, as illustrated below.

#### Masks, Lockdowns, Etc

The topics of *masks and lockdowns, etc* (which includes social distancing) are discussed in the context of vaccines in our data set. Frequently raised issues in antivax tweets included (1) why a vaccine is needed if masks, lockdowns, and social distancing are effective; (2) why masks are necessary if vaccines are effective; (3) that masks and lockdowns violate basic human rights and are used to control the population and create a need for vaccination; and (4) how leaders and figureheads who were against masks are now in priority queues for vaccines.

In contrast, the provax tweets were overwhelmingly supportive of masks, lockdowns, and social distancing, discussing their importance in controlling the spread of the virus and saving lives in the prevaccine period. For antivax tweets, the most significant peak in *masks and lockdowns* in Figure 3A came immediately after the Sputnik announcement (see earlier). The only significant peak in the provax *masks and lockdowns* topic in Figure 3B is due to the following viral tweet on June 2, 2020:

Curfew for black lives matter but none for coronavirus.....black people can’t be more dangerous than an airborne virus with no vaccine.

#### Pfizer and Moderna

Most of the activity on this topic is in the postvaccine period, and many peaks in Figure 3A coincided well with relevant current events, including the Sputnik vaccine announcement and approval of the Pfizer and Moderna vaccines in the United Kingdom and the United States.

#### Big Pharma

Pandemic profiteering by pharmaceutical companies is a major issue discussed in antivax tweets under *big pharma*. They include a discussion of significant increases in profit for Pfizer (and its partner company BioNTech) and Moderna [111,112] instead of supporting open licenses to boost vaccination delivery in countries with low uptake rates. The famous reply by the Polio vaccine inventor Jonas Salk, “could you patent the sun?” in response to why he did not patent his invention was quoted in >2000 tweets. Profiteering is generally frowned upon in both antivax and provax tweets, although provax tweets are mostly positive about the role of pharmaceutical companies in the development of COVID-19 vaccines. Some antivax tweets, however, suggested that the pandemic was purposely created for business and power-grabbing opportunities. For example, the *big pharma* antivax tweets peaked on September 24, 2020, because of the news about the United Kingdom’s chief scientific adviser’s £600,000 (US $762,000) worth of shares in GlaxoSmithKline [113].

#### Bill Gates

The discussion about Bill Gates is prominent in the prevaccine period, with almost 96.15% (3697/3845) of the antivax tweets on April 9, 2020, being on this topic (see *Bill Gates* in Figure 3A). This activity caused a noticeable spike in total antivax tweets (Figure 1A), and a likely driver was the appearance of Bill Gates on Trevor Noah’s The Daily Show on April 2, 2020. The second peak was due to a surge in tweets on August 8, 2020, after Bill and Melinda Gates funded US $150 million to the Serum Institute of India and the GAVI vaccine alliance for vaccine development [114]. News, which appeared in the tabloid press, about Elon Musk calling Bill Gates a “knucklehead” [115], was shared many times and caused a peak on September 29, 2020.

In addition to these peaks, many antivax tweets covered various conspiracy theories, such as the pandemic being orchestrated to create a market for vaccines, often linked to Gates’ 2015 TED Talk on the next epidemic outbreak or the global public health efforts supported by the Bill and Melinda Gates Foundation. Other conspiracies, such as *microchip in a vaccine* and *eugenics*, were also directed to Bill Gates and his foundation’s support and interests in e-vaccine cards. His subsequent interviews and statements to clarify his role in vaccine development appeared only to amplify antivax tweet rhetoric.

There was little interest from the provax side in this topic (399,560/37,044,507, 1.08%). Provax tweets largely shared news and updates about funding from the Bill and Melinda Gates Foundation and efforts in vaccine development. Some tweets also ridiculed the conspiracies associated with Bill Gates, especially about microchips in the vaccine.

### The Provax Tweets

Of the top 10 provax topics shown in Figure 3B, *vaccine development* was dominant throughout the study period. More than 51.26% (18,989,506/37,044,507) of tweets discussed this topic, including updates from clinical trials, news about the efforts of governments to fund research and acquire vaccines for their people, vaccine rollout details, scientific information about vaccines—especially messenger RNA technology and news about public leaders and celebrities making donations and publicly receiving a vaccine. The topic plot shows various peaks that can be attributed to a combination of different causes, such as press releases on clinical trials, including trial results and issues, important vaccine announcements, updates on approval procedures, and the launch of various contact tracing apps. We observed peaks in *vaccine development* on August 12, 2020, and from December 7 to 20, 2020 (for the particular vaccine-related events, see *The Antivax Tweets* section). One peak is attributed to the start of the Indian vaccination program on January 16, 2021. The US Presidential Debates were also referenced in the context *of vaccine development*, especially in the weeks leading up to the elections.

The topic *debunking antivax conspiracies* also shows multiple peaks from December 14 to 20, 2020, which is because of a viral meme mocking antivax concerns regarding the ingredients in vaccines. This topic mostly contains humorous tweets that make fun of antivax conspiracies, such as vaccines containing microchips or governments attempting to kill people using vaccines.

The topic of *provax (general)* is the second most popular provax topic with 8.95% (3,315,904/37,044,507) of tweets and includes positive statements about the COVID-19 vaccines and vaccine in general, such as people posting after receiving the COVID-19 vaccine, sharing updates about vaccine rollout and eligibility, and encouraging other people to get the vaccine. There is a significant overlap between the topics of *vaccine development*, *distribution*, *setbacks and issues*, *access to vaccine*, *provax (general)*, and *debunking antivax conspiracies*. Altogether, they defined almost 71.87% (26,623,517/37,044,507) of the provax tweets. The rest of the provax topics are discussed in Multimedia Appendix 1.

### Memes

Memes or humorous tweets, with occasional variations, were among the most frequently retweeted in both the antivax and provax groups. The results are summarized in Table 1. These funny messages, although carrying minimal arguments or information, are perhaps more memorable and shareable. Of the top 10 most retweeted antivax tweets, 7 were jokes or memes with multiple variants, which constituted almost 5% of the total volume of antivax tweets. We also examined the top 15 most retweeted provax tweets (the top 10 had only 2 memes) and found 5 different jokes and memes. Altogether, these accounted for 1.3% of the total provax tweets.

Determining whether memes and humor were deliberate attempts to advance the antivaccine discourse is an important question, but it is beyond the scope of this paper. What is perhaps remarkable is that based on the timeline identified in Table 1, there appears to be a reactionary tête-à-tête between antivax and provax memes, triggered by a sequence of news events around vaccine approval and development (see events 3-5 in *The Antivax Tweets*).

**Table 1:** Memes among high-frequency antivax and provax tweets.

Original tweet and example variations		Start	Variants, n	Tweets, n (%)
**Antivax (from top 10 tweets; n=37,047,378)**				
	“Me after taking the Covid-19 vaccine [funny picture]^a^,” “Me and the girls once we get the vaccine [funny picture],” “Me and the boys when we get the Covid vaccination [funny picture],” “snoop dogg after the vaccine [funny picture],” etc	November 9, 2020	103	245,658 (2.3)
	“When that vaccine hits [funny picture],” “The gang and I after that vaccine hits [funny picture],” “Me and the girls when the vaccine hits [funny picture],” etc	December 8, 2020	34	71,438 (0.7)
	“He is not lying, this is me before and after the vaccine. not enough people are speaking up about this [funny picture]”	January 17, 2021	1	38,492 (0.4)
	“*I Am Legend* was set in 2021... the zombie apocalypse was because of the failed vaccine.....”	January 14, 2021	1	38,355 (0.4)
	“i just saw^b^... homeless guys giving each other the covid vaccine under a bridge. what a caring community we live in,” “just saw 4 homeless men giving each other the covid vaccine under a bridge,” and “what a caring community we live in”	January 13, 2021	2	38,331 (0.4)
	“why they warming my vaccine in a spoon,” “how come they heated my vaccine up in a spoon?,” etc	January 18, 2021	3	37,510 (0.4)
	“This is the laboratory that invented the coronavirus vaccine [funny picture],” “This is what is wrong with the AZ vaccine,” “This is the vaccine [funny picture],” etc	November 11, 2020	17	35,857 (0.3)
**Provax (from top 15 tweets; n=37,047,378)**				
	“You eat sausages your whole life but you refuse vaccine because you do not know what’s in it,” “if u eat jack in the box tacos do not worry about what’s in the covid vaccine,” “If you ate these as a kid. You do not have to worry about the vaccine,” etc	November 20, 2020	254	245,051 (0.7)
	“n^****^s is more scared of the vaccine than the virus,” “How y’all scared of the vaccine but not scared of Covid,” etc	December 5, 2020	4	75,222 (0.2)
	“My boyfriend got his covid vaccine yesterday and I can tell you the most prominent side effect is the inability to shut up about getting the covid vac”	December 16, 2020	1	72,117 (0.2)
	“We gotta get this God damn vaccine, when we get the vaccine”	November 25, 2020	2	45,752 (0.1)
	“Y’all swear the government need a vaccine to kill y’all like Burger King do not sell 10 nuggets for $1.50”	December 19, 2020	1	37,336 (0.1)

^a^See Multimedia Appendix 1 for examples of funny pictures referred here.

^b^Ellipsis at this instance represents a hidden number.

### Genuine Concerns Versus Falsehoods in Antivax Discourse

**Figure 4:**
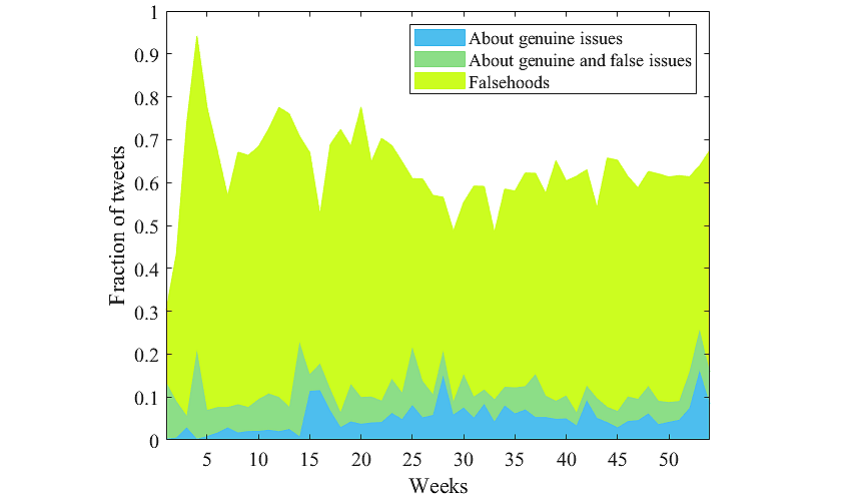
Besides the conspiracies about messenger RNA vaccine altering DNA, a lot of misinformation and falsehoods found their way into all antivax topics, such as side effects and vaccine mandate. We filter the tweets that discussed some genuine issues, although they may still contain misinformation. The "about genuine issues" plot shows that only 10% to 15% of the antivax tweets referred to some legitimate concerns. The "falsehoods" plot tracks the tweets about fictitious or spurious issues. The "about genuine and false issues" plot shows the fraction of tweets that have keywords related to both genuine and false issues.

A key problem of interest is to understand the contribution of misinformation and falsehoods toward the antivax discussion. To investigate, we separated the keywords and key phrases used for antivax topic classification into three groups: (1) genuine issues, (2) falsehood, and (3) neither. Then, we counted the number of antivax tweets with topics appearing in the first 2 groups, as the *neither* group could also be put together with the antivax tweets not classified into any topic. It is possible that one tweet can be classified into multiple groups, as shown by the plot of *About genuine and false issues* in Figure 4, as the tweet may contain keywords related to genuine issues and falsehoods.

The topics of genuine or legitimate concerns were identified by consensus among the authors, as detailed in Multimedia Appendix 1. We consider these topics genuine concerns, as they offer some grounds for reasoned debate and argumentation, as opposed to demonstrably false claims or fictitious issues where no reasoned discussion is possible.

The genuine concerns are (see Multimedia Appendix 1 for details) as follows:

1. Mandatory vaccines and loss of freedoms
2. Fast-tracked vaccine
3. Historical issues with vaccines and clinical trials
4. Pharmaceutical companies profiteering
5. General vaccine side effects
6. Blood clots after receiving the Astra Zeneca vaccine
7. Waning immunity and virus variants
8. Administrative mismanagement
9. Animal abuse during vaccine development.

Conversely, topics found under falsehoods include conspiracies, antivax memes, and tweets about fictitious issues. The conspiracies we identified were *experimental mRNA or altering DNA*, *vaccine contains microchip*, *metal or nanoparticles in vaccine*, *cells from aborted babies in vaccine*, *fertility issues*, *Robert F Kennedy* (as a figurehead in the vaccine skepticism movement), *related to flu vaccine*, *eugenics*, *vax makes you gay*, and *QAnon*. Multimedia Appendix 1 provides a detailed breakdown of this group of 10 *conspiracies*. Fictitious issues include blaming COVID-19 vaccines for the deaths of 23 elderly people in Norway, claiming that COVID-19 fatality rates were lower in comparison with influenza, claiming that COVID-19 has naturally diminished without a vaccine, or claiming that there was no point to having vaccines. The tweets that are not classified into either *genuine issues* or *falsehood* are typically simple statements, such as “I do not want a vaccine,” or accusations at public leaders because of something they said or did, discussions about alternative treatments being a better choice than a vaccine, and also some tweets that are not classified because of lack of suitable keywords.

Figure 4 shows the fraction of tweets in each group for each week of the year. Only 10% to 15% of antivax tweets contained some genuine issues, whereas a large portion was based on fictitious issues and falsehoods (although *conspiracies* accounted for only 709,877/10,566,679, 6.71% of the antivax tweets). Approximately 38.09% (4,025,547/10,566,679) of the yearlong antivax tweets belonged to neither of the groups. We remark that our keyword-based classification cannot distinguish between well-reasoned and poorly reasoned tweets about a genuine topic. Many tweets identified as discussing a *genuine concern* used excessive exaggeration or presented information about true facts or events out of context, such as the following tweet:

The last rushed vaccine cost the government £60M in injury compensation. How long will it be when we hear about the side effects of a COVID-19 vaccine already rolled out to millions? Humanity has lost it’s way, a poisonous concoction is “for our safety.”

Nonetheless, there are some well-reasoned tweets in this group, such as:

...Take the jab or lose your job: Medical journal calls for a MANDATORY Covid vaccine, says “noncompliance should incur a penalty.”

From Figure 4, falsehoods clearly dominate the topics discussed in antivax tweets, although there is a slight decline toward the end of the observation period. Although our findings around dual-stance users suggested that users on Twitter are not as polarized around COVID-19 vaccines as one may initially assume, the significant number of falsehoods found in antivax tweets confirms that addressing web-based misinformation around vaccines remains a critical challenge.

### The Dual-Stance Users

An example of antivax and provax tweets from a dual-stance user is provided below to highlight the complex nature of the content produced by many dual-stance users:

I work in pharma and I’m not getting it either. I know how to read journal articles and clinical trial data. The risk versus potential reward isn’t worth it, especially with the unknowns of an RNA vaccine. But I’m still called a conspiracy theorist.

As the virus gets worse, people get more nonchalant about it. It makes no sense. We have vaccines. There’s a light at the end of the tunnel. Be safe for another few months and we’ll be out of this. We can all be heroes just by being careful. Saving lives has never been easier!

Finding a sizable cohort of such users who engage in both provax and antivax content was unexpected. Even when we consider noise in stance detection, there is still a significantly large dual-stance user group (see details in Multimedia Appendix 1). In fact, they are the most prolific contributors, who sent most of the provax and antivax tweets, that is, 66.04% (24,463,708/37,044,507) of the provax tweets and 85.24% (9,007,481/10,566,679) of the antivax tweets (see details in Multimedia Appendix 1). Among dual-stance users, close to 60.24% (1,140,448/1,893,231) have tweeted more provax tweets than antivax tweets, 22.53% (426,568/1,893,231) have tweeted equal numbers of both types of tweets, and approximately 17.23% (326,215/1,893,231) have posted more antivax tweets than provax tweets (see Multimedia Appendix 1 for details).

**Figure 5:**
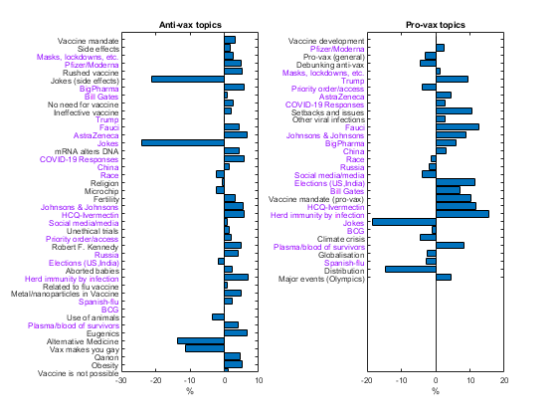
The normalized difference in actual versus expected (according to the population ratio) contributions of dual-stance users for each topic. Positive and negative values indicate that dual-stance users contributed more or less than expected, respectively, in relation to the contributions from users who only tweeted about antivax or provax topics. Dual-stance users are relatively more active in most of the discussion topics. Interestingly, jokes are more popular among pure antivax and pure provax users. BCG: Bacillus Calmette–Guérin vaccine; HCQ: Hydroxychloroquine medicine.

To characterize dual-stance users’ tweets relative to pure antivax and pure provax users, we computed the normalized difference between the actual and expected contributions of dual-stance users in each topic, as shown in Figure 5. The expected contribution of dual-stance users to a provax, or antivax, topic is calculated by multiplying the number of topic tweets by the ratio of dual-stance provax, or antivax, tweets to total provax, or antivax, tweets, that is, 0.66, or 0.85 respectively. An excess contribution by dual-stance users corresponds to a lesser contribution, by an equal amount, from the pure provax or antivax users. The most striking observations are (1) dual-stance users are more active than both pure provax and antivax users in most of the discussion topics and (2) jokes are mostly popular with both pure provax and antivax users (see Multimedia Appendix 1 for more details).

A number of dual-stance users posted updates about vaccine development but also shared concerns about the vaccine, such as vaccines being fast-tracked, long-term side effects of vaccines being unknown, vaccines being mandatory, and enormous vaccine-driven profits for pharmaceutical companies. This can be observed from the excess contribution shown in Figure 5 for the most discussed provax and antivax topics. Note that small excess contributions are significant if we consider the number of tweets classified in these topics. However, not all concerns expressed by these users are genuine, as the antivax discourse is heavily packed with falsehood (Figure 4). Considering the uncertainties and highly stressful circumstances of our study period, the expression of concerns and worries about the COVID-19 vaccines is probably understandable, but there is also proliferation of misinformation from this cohort (slightly less when compared with pure antivax users; details in Multimedia Appendix 1), who are supposedly exposed to both sides of the debate.

At the first glance, dual-stance users—those who send provax and antivax tweets—may be puzzling. This may even be counterintuitive from the stereotypical view of Twitter users as ideologically motivated hardliners with fixed views. However, the circumstances of the pandemic and the psychology of the people in it suggest that there may be a number of people who oscillate between pro- and antistances. The COVID-19 pandemic has been an unprecedented threat to humanity. Its gravity was unclear, and vaccines and their effectiveness in combating infections were also uncertain. Under such high levels of uncertainty, it is not surprising that people’s views on vaccination are swayed by whatever information may be available and salient at the time. Indeed, ever since the early days of attitude change research, psychological experiments have shown that at least some, if not all, of ordinary people’s attitudes and opinions can change relatively easily [116-118]. Thus, the changing stances of dual-stance users may reflect the volatility and uncertainty of the COVID-19 pandemic.

## Discussion

### Principal Findings

In this paper, we used natural language processing–based stance detection and composite topic modeling to examine a Twitter data set containing 75 million English tweets relating to COVID-19 vaccines, classifying tweets into *provax* and *antivax* stances, and exploring leading factors behind each stance. We examined the unique user IDs in each group and found a power law distribution describing the number of users with a given number of tweets, indicating a significant contribution to the discussion from a small number of users (see Multimedia Appendix 1 for details). Moreover, we found close to 2 million dual-stance users who had tweeted both antivax and provax tweets. Our topic analysis revealed the core topics discussed by the *antivax* and *provax* tweets, including a set of common topics. Antivax discussion was spread across multiple topics, such as vaccine safety and mandates. Falsehoods featured significantly more than discussions in reference to genuine concerns in almost all antivax discourses. In contrast, provax discussions centered primarily on vaccine development. Peak tweet activity often appeared to coincide with a key COVID-19 current event or media report, whereas memes and jokes featured heavily in the most popular retweeted messages.

### Comparison With Prior Work

A growing body of literature has analyzed social media posts, particularly tweets related to COVID-19 vaccines. Here, we compared our work with 29 significant Twitter studies [2-7,18,20,21,23-26,119-134]. Unsupervised methods, such as sentiment analysis and topic modeling, are the most popular methods used to classify and analyze tweets; 11 studies used some combination of sentiment analysis, emotion analysis, and topic modeling [3,6,20,21,24,124,126,128,130,131,134]. Most of these studies analyzed large tweet data sets comprising millions of tweets. In contrast, 6 studies conducted manual content analysis of a few thousand tweets [4,18,23,119,123,133], whereas another 6 papers used lexical and linguistic models, proprietary or otherwise, to detect vaccine hesitancy [7,20,25,121,129,132] using moderate-sized data sets (up to several hundred thousand tweets). Finally, a few papers classify hashtags [120,133] or URLs [2,126] to differentiate between antivax and provax tweets using large data sets with millions of tweets. Table 2 presents a summary of prior studies and their main contributions.

However, most of these studies used unsupervised learning methods, such as sentiment analysis and topic modeling, to indirectly infer the provax versus antivaccination stances expressed by tweets. In contrast, our study used a direct measure of vaccination stance. Stance detection, particularly with transformer models, is a superior method for classifying tweet stances, although it requires substantial amounts of labeled data to train the model. We accomplished a reasonably accurate stance classification of 75 million tweets using 46,176 tweets for the training, validation, and testing of the stance detection model. Hayawi et al [5] used transformer models with 15,000 labeled tweets for training but did not analyze tweet content but examined the efficacy of different stance detection tools. The polarization analysis in the study by Pierri et al [127] also used a Bidirectional Encoder Representations from Transformers transformer with 5000 labeled tweets to detect the stances of approximately 17,000 tweets. Closest to this study is the work in [128], which has used 1500 labeled tweets and detected stances of 15 million tweets from 3 distinct periods from January 2018 to December 2019, January to March 2020, and January to March 2021. They also identified 12 salient discussion topics. Our study only covered the pandemic period, in contrast to Bustos et al [128] but provided an in-depth and comprehensive analysis.

Our analyses suggest that neither sentiment analysis nor topic modeling provides accurate measures of tweet stances. First, sentiment scores by TextBlob were reported in Lamsal’s data set [27] and used in our study. However, according to our analysis, only 21% of the tweets in this data set with an antivax stance have a negative sentiment score, whereas 41% had a positive sentiment score. Interestingly, 21% of the provax tweets also had a negative sentiment score, whereas only 43% had a positive sentiment score. In other words, sentiments expressed in tweets do not agree with their stances taken in them. In addition, topic modeling approaches to stance prediction may misestimate the prevalence of antivax stances. Yousefinaghani et al [6] classified relatively more tweets as antivax than provax, and Gori et al [4] and Hou et al [18] have also reported relatively more antivax than provax tweets when they analyzed a couple of thousand randomly selected tweets. However, as reported earlier, our analyses found that provax tweets (37 million) far outnumbered antivax tweets (10 million). Furthermore, the topic of a tweet may not accurately indicate its stance. We found that the same topic was discussed from a provaccination perspective and also from an antivaccination perspective. Table 2 compares this study with existing studies in terms of its scope and main findings. This paper presents one of the most comprehensive studies analyzing vaccine-related tweets during the first year of the pandemic.

Our approach complements extant works that have explored the spread of misinformation over Twitter and other social media [17] by examining the key topics discussed and differentiated by stance. We also tracked the topic evolution over an entire year, again differentiated by stance, identifying topics that have a sustained presence in the Twitter discourse for both antivax and provax tweets (eg, *vaccine mandate*) and those topics that emerge and disappear (eg, *vax makes you gay*; see Multimedia Appendix 1)—this contrasts existing works which focus on specific topics or narrow timelines at perhaps a higher resolution [20,136]. Trust and safety-related topics, for example, *side effects*, *rushed vaccine*, featured prominently in the antivax tweets, which supports other work that identified safety and trust (in institutions and governments) as a key hurdle in addressing vaccine hesitancy [18,20,23]. Our stance detection approach also enabled the definitive identification of a set of *dual-stance* users who contributed a significant volume to both antivax and provax tweets, supporting existing findings [4,26,119-121,133].

**Table 2:** Summary of existing Twitter studies regarding COVID-19 vaccines (last row summarizes this paper).

Classification method			Number of Tweets		Study period		Main findings		References
**Unsupervised**									
	Sentiment or emotion analysis	20,000-2 million		2 months to 1 year		Mixed results about the prevalence of negative sentiment Main topics: safety, trust, side effects, vaccine rollout		[3,6,20,21,24,124,126,128,130,131,134,135]	
	Topic modeling	0.7-1.5 million		5-10 months		Up to 16 topics were found Most negative sentiment for AstraZeneca		[24,130,134]	
	Linguistic models	26 million		7 months		Ontological classification of antivaxxers		[121]	
	Community or coordination detection	7500-29 million		5-14 months		Communities have majority of users with one stance Coordinated activities detected in propagation of conspiracies		[119,122]	
	Keyword-based classification	0.3-1.8 million		3 months, 10 years (historical)		More antivax tweets for government, politics, and conspiracies		[7,132]	
**Supervised**									
	Manual coding	<10,000		2-14 months		Mixed reports about the prevalence of antivaxxers Major topics of antivax tweets Location-wise differences in topics		[4,18,23,119,123]	
	URL or hashtags	237,000-37 million		15 months		Causality between misinformation and vaccine uptake Stances were politically polarized Humans have more influence than bots		[2,120,127,133]	
	Proprietary tools	1000-91,473		1 week-9 months		Antivax persuasion is through anecdotes, humor, etc Safety and trust are main issues		[20,129]	
	Bidirectional Encoder Representations from Transformers transformer	17,000-15 million		4 months to 3.2 years		Vaccine sentiments are polarized across political ideologies. 12 topics are tracked		[5,25,26]	
	GPT	75 million		1 year		53 topics were found; provax prevails; main topics: (antivax) safety and mandate, (provax) vax development		This paper	

### Limitations

Nonetheless, our study has certain limitations that we hope to address in future studies. First, the Lamsal data set, from which we extracted the data that was analyzed, contains only English tweets; hence, our findings are most relevant to nations with English as the predominant language (eg, the United States, the United Kingdom, Australia, and Canada). We did not attempt to differentiate based on location, as Twitter geolocation data can be unreliable. It would be of interest to conduct similar stance detection-based analyses for tweets in other languages, perhaps using cross-lingual transfer learning techniques [137], or to extend the study period for another year to observe how vaccine uptake impacted and was being impacted by the vaccine discourse on Twitter. Second, we have only considered Twitter, and it would be of interest to apply our trained model to data sets from other social media platforms, such as Reddit. Third, we were not able to hydrate all tweet IDs from the Lamsal data set (eg, archived tweets by Donald Trump and their retweets were not visible after his Twitter account was banned). To ascertain that the trends observed in antivax and provax tweets are not sensitive to hydration loss, we also detected the stances of another data set collected at the University of Melbourne from February 2020 to July 2020. The results and details are provided in Multimedia Appendix 1.

Since early 2023, Twitter, Inc, has changed its policies regarding developers’ accounts, which in the past allowed academic researchers to access data on social media platforms. It is now prohibitively expensive to carry out similar research; although we have shared tweet IDs and associated stances on our GitHub page, it may not be possible for researchers to use the data from this study, as they may not be able to hydrate the tweets using the IDs. This will create nontrivial challenges for future efforts that aim to expand on our work or explore other aspects of the data, although such challenges are not insurmountable. Moreover, Twitter Inc indicated a possible policy change in the future to provide free or low-cost accounts for academic research, and we hope that this incredibly important resource could still be used to increase our understanding of the complex vaccine debate.

### Conclusions and Future Work

A thorough account of the COVID-19 vaccine discourse in the first year of the pandemic is presented in this paper and Multimedia Appendix 1. Although provax content is mostly about vaccine development updates, the antivax tweets discussed a broad range of topics, including vaccine safety and mandate, although they rarely touch upon some genuine concern and instead contained a large dose of misinformation. Contrary to the popular perception that users driving the antivax rhetoric on social media are vocal, hardcore, and probably homophilic, we found that most antivax tweets were posted by users who also posted in favor of COVID-19 vaccines. Not only we did not find evidence of the prevalence of antivax expression on Twitter, but our findings do not support the idea of polarization of COVID-19 vaccine discourse. This dual-stance user conundrum, which dominated the discourse, may reflect the volatility and uncertainty of the COVID-19 situation.

Our study lays the groundwork for several promising directions for future research. Obviously, it would be of interest to extend our analysis to cover the second year of the COVID-19 pandemic, as our observation period ended as COVID-19 vaccine roll-outs accelerated in many countries. A more in-depth analysis of users, especially dual-stance users who contributed significantly to the overall Twitter discussion environment, would complement the current focus on topic modeling. A closer examination of how antivax and provax topics interact, including determining causality relationships, would also be of interest, for example, identifying whether peak activity in a provax topic led to peak activity in some antivax topics.

## Appendix

Multimedia Appendix 1 Additional details regarding the data set, analysis of the dual-stance user cohort, and details of less significant topics.

## Abbreviations

AZ: AstraZeneca
FDA: US Food and Drug Administration
GS-DMM: Gibbs Sampling Dirichlet Multinomial Mixture
LDA: latent Dirichlet allocation
RQ: research question

## Data Availability

The code used and the data generated in the simulations are available on the internet [138].
