# Supplementary material for: Topics in Antivax and Provax Discourse: Yearlong Synoptic Study of COVID-19 Vaccine Tweets
Source: J Med Internet Res. 2023 Aug 8;25:e45069. doi: 10.2196/45069 (PMC10411425; doi:10.2196/45069)
Supplement: Multimedia Appendix 1 [file jmir_v25i1e45069_app1.docx]

Multimedia Appendix 1 for

Topics in Anti-vax and Pro-vax discourse: An Yearlong Synoptic Study of COVID-19 Vaccine Tweets

# Zainab Zaidi* and Mengbin Ye, Fergus John Samon, Abdisalam Jama, Binduja Gopalakrishnan, Chenhao Gu, Shanika Karunasekera, Jamie Evans, Yoshihisa Kashima1

*Corresponding author: [Zainab.RaziaZaidi@unimelb.edu.au](mailto:Zainab.RaziaZaidi@unimelb.edu.au)

The Appendix 1 is organised as follows. In Section [1](#_bookmark0), we present the figure of COVID-19 related tweets’ time-series. Section [2](#_bookmark1) discusses the generic tweeting behaviour observed in the study and Section [3](#_bookmark3) talks about the contribution of dual-stance users. In Section [4](#_bookmark3), we present the table of discussion topics and figures to show yearlong evolution of anti-vax and pro-vax topics. Section [5](#_bookmark8) contains the explanation of genuine concerns discussed in the main text, Section [6](#_bookmark9) presents examples of anti-vax memes with funny pictures, and Section [7](#_bookmark10) has a selection of tweets comprised of false reports and incidents of adverse reactions which never happened. Sections [8](#_bookmark15), [9](#_bookmark33), and [10](#_bookmark35) present the lesser discussed anti-vax, common, and pro-vax topics, respectively. Section [11](#_bookmark35) presents some interesting themes from the vaccine discourse, Section 12 discusses the second dataset we analysed which was collected at University of Melbourne, and Section 13 has the annotation guidelines used to label the tweets as favour, against, or none with respect to the topic ‘vaccine hesitancy’.

#
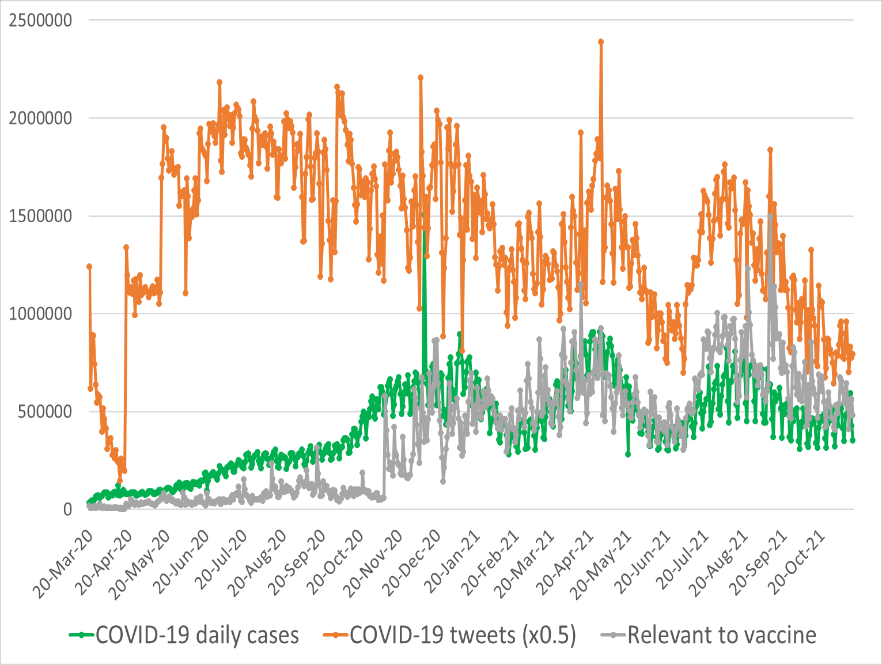
Tweets’ Time-series

**Figure 1.** COVID-19 tweets (orange), rescaled by 0.5 to fit the chart, from Lamsal dataset [27] are further filtered with vaccine related keywords (grey). The temporal dynamics of both COVID-19 tweets and COVID-19 vaccine tweets, have a strong correlation with the global daily COVID-19 infections (green) — infection data is from [36].

Figure [1](#_bookmark2) shows the time-series of COVID-19 tweets and the tweets filtered by vaccine-related keywords. We have superimposed them over the global daily COVID-19 infections data collected by Johns Hopkins University [36]. Three distinct waves of COVID-19 global infections are clearly visible in Figure [1](#_bookmark2) and the time series for both sets of tweets closely follow the trend of the infection waves. This yearlong study, from March 20, 2020 to March 23, 2021, completely covers the first wave. The two significant changes in total COVID-19 tweets in Figure [1](#_bookmark2) during April and May 2020, are due to changes in tweet collection process [27].

# Power Law Distribution

COVID-19 related tweets are classified into anti-vax and pro-vax tweets using stance detection tool based on OpenAI’s GPT model [28]. Figure 2a shows the number of users with a given number of tweets during the study period. Both anti-vax and pro-vax classes follow power law distribution with similar exponent. This observation conforms to the previous studies [37], [38], which found user aggregate behaviour over Twitter often follows power law distributions. Figure 2b plots separately the tweets/user statistics for dual-stance users from pure anti-vax and pure pro-vax users, which also shows that dual-stance users are more active than those who posted only single stance tweets.

#
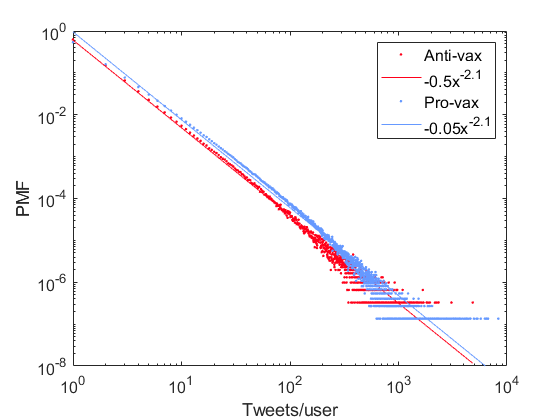

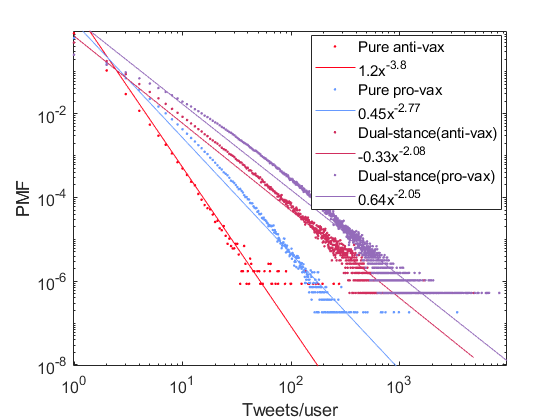


(a)

(b)

**Figure 2.** (a) The distribution or PMF (Probability Mass Function) of users with a given number of tweets, over a log scale. Tweets/user followed a power law αx^γ^ distribution for both classes (also shown with straight lines), i.e., there were many users with few tweets and there were a small number of users with many tweets in both groups. (b) Tweet/user distributions and associated power law distributions drawn separately for dual-stance users for anti-vax and pro-vax tweets and pure anti-vax and pure pro-vax users. Dual-stance users had more users with many tweets than the pure anti-vax and pure pro-vax groups.

# Contribution of Dual-stance Users

Figure [3a](#_bookmark4) shows the normalised histogram, i.e., the value in each bin is divided by the total population, of dual-stance users with specific proportion of pro-vax tweets, i.e.,

*n_p_*

*n_a_* + *n_p_ ,*

where *n_p_* (*n_a_*) is the number of pro-vax (anti-vax) tweets for each user. As seen in Figure [3a](#_bookmark4), the dual-stance cohort contains users with balanced posts in both stances (close to 17%) and also with dominant anti-vax or pro-vax expression. Almost 60% of the users are counted on the right half of Figure [3a](#_bookmark4), which means they have more pro-vax tweets than anti-vax tweets. The users on the left side of the Figure [3a](#_bookmark4) are 23% of the total dual-stance users and contributed more anti-vax tweets than pro-vax tweets.

In order to ascertain that the finding of dual-stance user cohort is not a mere artefact of stance detection noise, we looked into the probability *p_i_* that a user *i* detected as a dual-stance user with *n_a_* anti-vax tweets and *n_p_* pro-vax tweets is in fact dual-stance or not, assuming independent detection error from one tweet to another, i.e.,

*p_i_* = 1 *−* (1 *−α_a_*)*^na^ −* (1 *−α_p_*)*^np^* + (1 *−α_a_*)*^na^* (1 *−α_p_*)*^np^ ,* (1)

where *α_a_* (*α_p_*) is the precision of stance detection tool for anti-vax (pro-vax) class. For our dataset *α_a_* is in the range of 052-0.92 and *α_p_* is in between 0.68-0.95 calculated using test datasets. The effective size of the dual-stance cohort *N_e_ _f_ _f_* can be calculated as

*N*

*N_e_ _f_ _f_* = ∑ 1*.p_i_,* (2)

*i*=1

where *N* is the number of detected dual-stance user. For the precision of stance detection method given above, *N_e_ _f_ _f_* is from 1,212,327 to 1,791,219. Even with the stance detection noise, we can safely conclude that there are significantly large number of users who have tweeted anti-vax and pro-vax tweets.

As given in Figure [3c](#_bookmark6), the dual-stance users contributed 85% of the anti-vax tweets for the yearlong study period, although, they were 62% (1,893,232 out of total 3,065,069) of the anti-vax users. On the other hand, they contributed more than half of the pro-vax tweets (66%) during the study period while they were only a quarter of pro-vax users (7,465,178 total pro-vax users). The figure shows the fractional contribution of dual-stance users in anti-vax and pro-vax conversation for each day within our study period.

For each anti-vax or pro-vax topic, expected contribution from dual-stance users will be in proportion to their contribution in overall anti-vax or pro-vax tweets. For example, if the ratio between the anti-vax tweets by dual-stance users to all anti-vax tweets is 0.85, then the expected tweets classified into anti-vax topic *i* by dual-stance users is,

*E_c_*(*i*) = *T* (*i*) *×* 0*.*85*.* (3)

where *T* (*i*) is the total anti-vax tweets classified into the topic *i*. The normalised differences in observed *A_c_*(*i*) and expected

*E_c_*(*i*) tweet count for dual-stance users, as reported in the main text, is

*A_c_*(*i*) *−E_c_*(*i*)

*T* (*i*) *.* (4)

Normalised differences between observed and expected contribution of dual-stance users for pro-vax topics are also calculated in similar manner. In Figure [3b](#_bookmark5), the normalised difference between the actual and expected contribution of dual-stance users towards tweets about genuine concerns, containing falsehoods, and also tweets which are not classified into either is shown. Dual-stance users are talking slightly more about genuine issues, and even slightly less about falsehoods. However, their engagement with misinformation and falsehood is still worrisome, as they seemed to have exposure to both sides of the debate.

**(a) (b)**

**(c)**

**Figure 3.** (a) The normalised histogram of dual-stance users with specific ratio of pro-vax tweets to total pro-vax and anti-vax tweets. (b) The normalised difference in actual versus expected (according to the population ratio) contributions of dual-stance users towards anti-vax tweets about genuine concerns, containing falsehoods, and the ones not classified into either. (c) The fractional contribution of dual-stance, pure anti-vax, and pure pro-vax users in daily anti-vax and pro-vax tweets for the yearlong study period. The small white spaces in both figures represent the tweets where users were not identified.

# Table and Yearlong Evolution of All Topics

Table [1](#_bookmark7) groups the topics under relevant themes. Table [1](#_bookmark7) also shows the percentages of tweets associated to each of the topics. Percentages in red colour are calculated from the anti-vax class and blue colour is associated to the pro-vax class. Since the topics are not mutually exclusive and one tweet can be assigned to multiple topics, the percentage contribution of the umbrella themes, such as, safety, issues about vaccine, or conspiracies, etc., is not necessarily equal to the sum of individual contributions of sub-topics under that theme. Rather, the percentage contributions of the umbrella themes are calculated using the union of all relevant keywords/key-phrases. All topics are discussed in detail either later in this document, or in the main text.

**Table 1.** COVID-19 Vaccine Discussion Topics.

| Anti-vax | 1- Issue about vaccine (35%):  1.1- Safety (29.5%):  1.1.1- Side effects (17.9%)  1.1.2- Rushed vaccine (7.4%)  1.1.3- Jokes regarding side effects (6.5%)  1.2- Efficacy (7.3%):  1.2.1- No need for vaccine (4%)  1.2.2- Vaccine is not possible (0.03%)  1.2.2- Ineffective vaccine (3.5%)  1.2.2- Effect of obesity (0.05%) | 2- Conspiracies (6.6%):  2.1- Microchip (1.4%)  2.2- Metal in vaccine (0.3%)  2.3- mRNA is altering DNA (1.9%) 2.4- Vaccine can make you gay (0.1%) 2.5- Fertility (1.3%)  2.6- Aborted babies (0.6%)  2.7- Related to flu vaccine (0.4%)  2.8- QAnon (0.1%)  2.9- Robert F. Kennedy (0.9%)  2.10- Eugenics (0.1%) | 1. Alternative COVID-19 cures (1.6%)   3.1- Alternative medicine (0.1%)  3.2- Religion (1.5%)   1. Vaccine mandate/ control (17.9%) 2. Use of endangered animals (0.1%)   6 - Unethical trials (1%) |
| --- | --- | --- | --- |
| Common Topics | 1- Big Pharma (5.2%,1.9%)  2- Race (1.6%,1.7%)  3- Spanish flu (0.2%,0.08%)  4- Jokes (general) (2.1%,0.3%)  5- Elections (US, India) (0.7%,1.1%)  6- Social media/ MSM (1.2%,1.3%) | 7- COVID-19 responses (11.5%,12.1%):  7.1- Herd immunity through infection (0.4%,0.3%)  7.2- Masks, lockdown, etc. (9%,4.5%)  7.3- Misc. COVID responses (1.9%,3.2%)  7.4- Access to vaccine/ priority order (0.9%,4.5%) | 8- COVID-19 treatments (1.5%,0.7%):  8.1- Plasma/blood from survivors (0.1%,0.1%)  8.2- HCQ/Ivermectin (1.2%,0.4%)  8.3- BCG (0.1%,0.2%) |
|  | 9- People (9.8%,7.6%):  9.1- Bill Gates (4.3%,1.1%)  9.2- Trump (3.4%,4.5%)  9.3- Fauci (2.8%,2.4%) | 10- Vaccine candidates (12%,15.5%):  10.1- Pfizer/ Moderna (8.6%,10.5%)  10.2- AstraZeneca (2.6%,3.6%)  10.3- Johnsons & Johnsons (1.3%,2.3%) | 11- Countries (2.5%,3.2%):  11.1- China (1.8%,1.9%)  11.2- Russia (0.9%,1.6%) |
| Pro-vax | 1- Vaccine development/ distribution (52.8%):  1.1- Vaccine development (51.3%)  1.2- Setbacks and issues (2.9%)  1.3- Distribution (0.03%) | 1. Pro-vax (general) (9%) 2. Debunking anti-vax conspiracies (5.6%) 3. Other viral infections (2.8%) | 1. Major events (Olympics) (0.03%) 2. Globlisation (0.09%) 3. Climate crisis (0.1%) 4. Vaccine mandate (pro-vax) (0.7%) |

Vaccine mandate 2


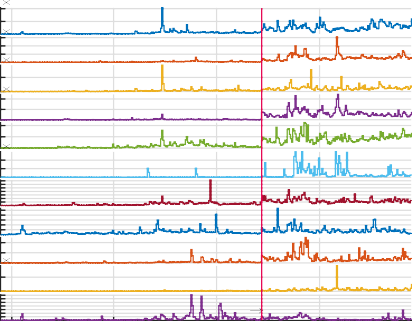


104

**Daily tweets for all anti-vax topics**

104

104

104

104

104

Pfizer announcement

Side effects 4

2

Masks, lockdowns, etc. 2

Pfizer/Moderna 1

Rushed vaccine 5000 Jokes (side effects) 2 Big Pharma 10000

1

6000

8000

Bill Gates

6000

4000

No need for vaccine 5000

Ineffective vaccine 2

Trump 10000

6000

Apr 2020 Jul 2020

**(a)**

Oct 2020

Jan 2021

2

Vaccine development 1.5


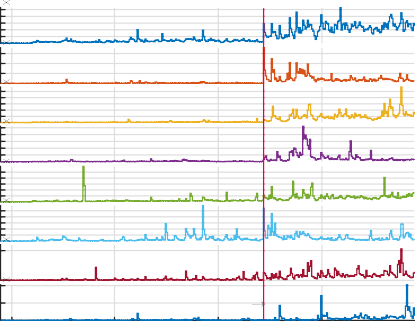


105

**Daily tweets for all pro-vax topics**

104

104

104

104

104

104

104

Pfizer announcement

1

0.5

15

Pfizer/Moderna 10

5

8

Pro-vax (general) 6

4

2

8

Debunking anti-vax conspiracies 6

4

2

5

4

Masks, lockdowns, etc. 3

2

1

4

Trump 3

2

1

5

Priority order/access

AstraZeneca 5

Apr 2020 Jul 2020 Oct 2020

**(b)**

Jan 2021

Fauci 10000


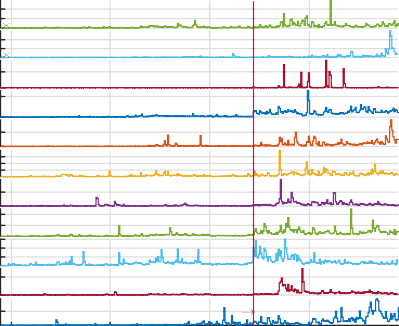


104

104

Pfizer announcement

5000

AstraZeneca 2

1

Jokes 2

5000

Experimental mRNA/altering DNA

Misc. COVID-19 Responses 5000

6000

China 4000

2000

Race 5000

Religion 2000

Microchip 2000

1000

Fertility 5000

Johnsons & Johnsons 2000

2

Misc. COVID-19 Responses 1.5


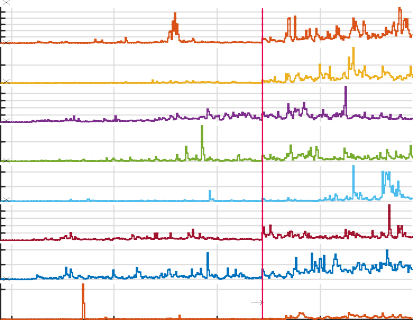


104

104

104

104

104

104

104

Pfizer announcement

1

0.5

Setbacks and issues 2

2

Other viral infections 1.5

1

0.5

Fauci 2

4

Johnsons & Johnsons 2

2

Big Pharma 1.5

1

0.5

10000

China 5000

5

Race

Apr 2020 Jul 2020 Oct 2020 Jan 2021

**(c)**

Apr 2020 Jul 2020 Oct 2020 Jan 2021

**(d)**

HCQ-Ivermectin 2000


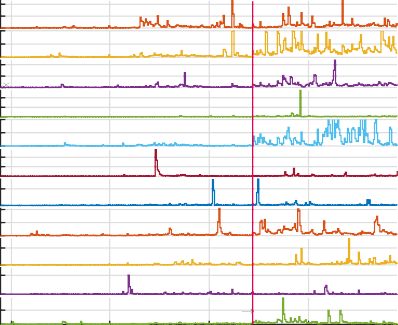


104

Pfizer announcement

Social media/media 1000

Unethical trials 2000

Priority order/access 2

1

Russia 5

4


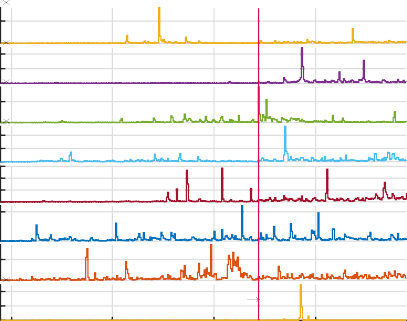


104

104

104

104

104

Pfizer announcement

Social media/media 2

Elections (US,India) 2

Robert F. Kennedy 1000

Russia 10000

5000

Elections (US,India) 5000

Aborted babies 1000

Herd immunity through infection 2000

Related to flu vaccine 5000

Metal/nanoparticles in Vaccine 1000

2

Bill Gates 1

Vaccine mandate (pro-vax) 10000

5000

5000

HCQ-Ivermectin

Herd immunity through infection 2000

4

Jokes 2

Apr 2020 Jul 2020 Oct 2020 Jan 2021

**(e)**

Apr 2020 Jul 2020 Oct 2020 Jan 2021

**(f)**

Spanish-flu 2000


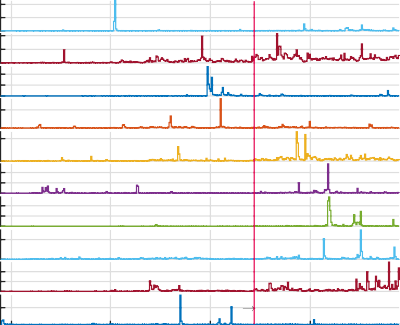


Pfizer announcement

BCG 200

2000

Use of animals 1000

Plasma/blood of survivors 1000

10000

BCG 5000


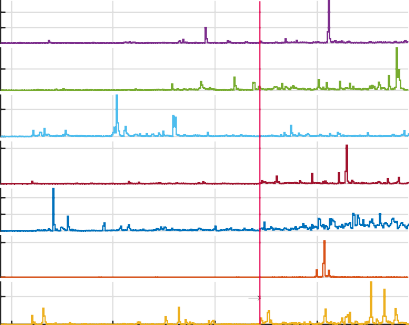


Pfizer announcement

Climate crisis 2000

Plasma/blood of survivors 2000

Eugenics

500

Globalisation

5000

Alternative Medicine 1000

500

Vax makes you gay 1000

500

Qanon 500

Obesity 200

100

Vaccine is not possible 500

1000

Spanish-flu 500

5000

Distribution

Major events (Olympics) 500

| Apr 2020 | Jul 2020 | Oct 2020 | Jan 2021 | Apr 2020 | Jul 2020 | Oct 2020 | Jan 2021 |
| --- | --- | --- | --- | --- | --- | --- | --- |
|  | **(g)** |  |  |  | **(h)** |  |  |

**Figure 4.** The yearlong evolution of anti-vax (a, c, e, g) and pro-vax topics (b, d, f, h), including the common topics (shown with violet colour) between anti-vax and pro-vax groups. Topics are sorted in descending order in terms of associated tweet volume. November 9, 2020 marks the start of post-vaccine period with Pfizer announcement.

Figures [4](#_bookmark12) (a, c, e, g) and [4](#_bookmark12) (b, d, f, h) show the yearlong evolution of all 43 anti-vax topics and 31 pro-vax topics from March 2020 till March 2021. The common topics are depicted with violet coloured labels. The fact that the topic ‘vaccine development’ is contained in an overwhelming proportion of pro-vax tweets is clear from Figure [4](#_bookmark12). November 9, 2020 marks the start of post-vaccine period with the Pfizer announcing their preliminary results [34], where a 10-fold increase in the tweet volume is observed. The data ranges for y-axis of stacked plots in Figure [4](#_bookmark12) are different from each other and are selected for clear visualisation of associated peaks.

# Explanation of Genuine Concerns

As discussed in the main article, we identify a set of issues within the anti-vax tweets, which we class as “genuine concerns”, and we now describe in further detail. These issues are selected based on a consensus among the authors. We identify these issues as genuine concerns because the discussion points in the tweets are at least plausible or demonstrated reasonable argument for why there is hesitancy in using the vaccines (as opposed to those topics which could be dismissed as they discussed clearly fabricated information or falsehoods).

1. **Mandatory vaccines and loss of freedoms** These tweets contained discussions about the possibility of COVID-19 vaccines becoming mandatory for everyone and that people will not be able to travel, shop, go to restaurants and other public places, or may lose their jobs if they refuse to be vaccinated.
2. **Fast-tracked vaccine** These tweets discussed the fact that all COVID-19 vaccines were fast-tracked with no long-term studies. Tweets pointed out the fact that such vaccines are being offered to the public under Emergency Use Authorisation (EUA).
3. **Historical issues with vaccines and clinical trials** These tweets pointed to a history of vaccine injuries and unethical practices in medical research and clinical trials, noting that such events cannot be ignored. Example events highlighted in the tweets included cases of contracting polio from contaminated oral vaccines in 1955 [39], the Tuskegee study [40], the story of Henrietta Lacks [41], and the Dengvaxia controversy [42], etc.
4. **Pharmaceutical companies profiteering** These tweets discussed issues around how pharmaceutical companies will earn tremendous wealth out of a vaccine which should have an open license for the common public good. The tweets also pointed out that pharmaceutical companies also have indemnity against legal action due to any side effects.
5. **General vaccine side effects** These tweets highlighted that vaccines, in general, can have the following potential side effects: 1- Bells’ palsy [43], 2- blood clots or thrombosis (TTS), 3-myocarditis/pericarditis, 4- hypoxia [44], 5- paradoxical immune response [45].
6. **Blood clots after AstraZeneca** These tweets focused on reports of blood clots (Guillain-Barré syndrome) after patients had received the AstraZeneca vaccine. Some tweets discussed the event in which a volunteer for the AstraZeneca clinical trials died in Brazil in October 2020 [46].
7. **Waning immunity and virus variants** Tweets that discussed this issue focused on the fact that immunity provided by vaccines wane over time, and virus mutations may make vaccine ineffective or less effective.
8. **Administrative mismanagement** Tweets concerning this issue discussed cases of administrative mismanagement, for example, reports of political pressure to approve vaccines before the 2020 US Presidential Elections [47], sudden changes in the recommended gap between doses in the UK in December 2020 [98], Australia’s procurement of only the AstraZeneca vaccine, etc.
9. **Animal abuse** This issue focused on the use of animals in testing and in vaccine development, which can endanger the animals’ existence. Top examples discussed in this respect are 1) use of horseshoe crab [48] in safety testing and 2) use of squalene, a natural oil found in sharks’ liver [49]. According to Politifact, however, none of the approved COVID-19 vaccines source squalene from sharks [50].

# Anti-vax Memes with Funny Pictures

Figure [5](#_bookmark13) contains some examples of anti-vax memes with funny pictures. The examples here are selected among those memes which did not contain any offensive and/or obscene content. Some anti-vax memes are now offline as well due to banned Twitter accounts.

**
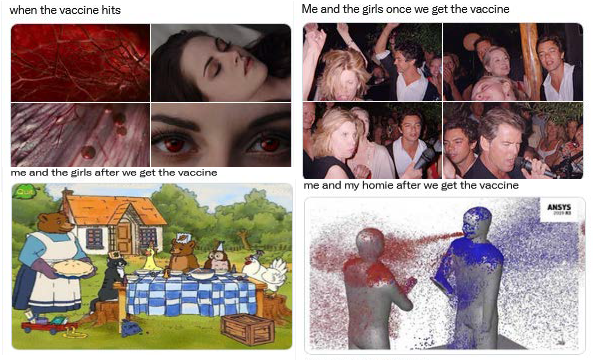
**

**Figure 5.** Some examples of anti-vax memes and jokes with funny pictures. Examples are selected among tweets which do not contain offensive and/or obscene material.

# Incidents reported in ‘side effects’ which never happened

Tweets classified under the anti-vax topic of ‘side effects’ contains misquoted and out-of-context reports about incidents of adverse reaction, such as the 23 deaths among elderly vaccinated Norwegians, and also reports of incidents which never happened. Some examples of such tweets are listed below,

1. Accusation that UK government blocked the Office for National Statistics from publishing side effects and deaths after taking covid vaccine were shared in 6642 tweets, although such a blocking action never occurred [51].
2. A tweet claimed that the FDA’s adverse reporting system reported 3 dozen cases of spontaneous miscarriages or stillbirths after administration of the Covid-19 vaccination. Retweeted 2461 times, this tweet has been debunked as a falsehood, and these claimed events never occurred [52].
3. A research paper published in Microbiology; Infectious Diseases, by J. Bart Classen is referred in a tweet which warns against the potential harmful impact of mRNA technology used in Pfizer and Moderna vaccines. This tweet was retweeted 1899 times, and it is an interesting example where published work from vaccine-sceptic scientists was shared to advance the anti-vax narrative [52]. The website of Robert F. Kennedy Jr. (https://childrenshealthdefense.org/) frequently posted research with anti-vax bias or even misreported research about vaccine, which were then shared many times by his followers over Twitter and other social media platforms. The research from this example tweet was also shared by the website of Robert Kennedy Jr.
4. A tweet, which was retweeted 1670 times, claimed that death is a side-effect of a vaccine which protects from a disease with 99% survival rate. The claim of 99% is totally wrong, as also discussed later in the section about conspiracies.
5. Bill Gates’ talk about the need of indemnity laws for pharmaceutical companies is presented as he wants to protect himself from liability while he pushes a vaccine with detrimental side effects on the millions of people. This tweet was shared 1606 times.
6. In a tweet, NHS England is cited as a source for reports of deaths in the 80+ population, since the vaccine roll out. This tweet was posted 1477 times and is false according to the UK’s Office of National Statistic’s report [53].
7. A viral tweet reported a family member taken to hospital with heart issues after taking the COVID-19 vaccine. It is difficult to verify the claim of this tweet, but it is an example of many similar tweets where one personal incident is reported and then the tweet is retweeted thousands of times. This tweet was posted 4876 times.

# Lesser Discussed Anti-vax Topics

The two sub-topics under the theme of ‘vaccine efficacy’: ‘**vaccine is not possible**’ and ‘**effect of obesity**’ are found in less than 1% of tweets in total. The concerns that no vaccine has ever been made before for coronaviruses, and that obesity may make vaccines less effective were the major subjects discussed under these topics, but the combined total of tweet assigned to them is under 10,000 for the whole study period.

The topic of ‘Alternative COVID-19 cures’ has only 1.6% anti-vax tweets assigned to it, with 1.5% tweets under ‘**religion**’ and only 0.1% for ‘**alternative medicine**’, where herbal medicines and home remedies are discussed. Historically, religiosity is found to be one of the major ideological antecedent for vaccine skepticism [54]. We also found similar moral concerns about the vaccine ingredients in the context of COVID-19 vaccine as well. The discussion ranged from Kanye West’s term ‘mark of the beast’ (which saw a peak on July 8, 2020) for micro-chipped vaccine to moral implications because of cell lines from past abortions [8]. Both issues are discussed in detail later in the context of the conspiracies. Pig gelatin and cow products were the main point of concern for Muslim and Hindu religious communities [55] we also found a couple of hundred tweets in our dataset talking about them. A survey by The Guardian, in November 2020, found that religious communities were also less likely to get vaccinated [56] (partly responsible for a peak in topic plot on November 19, 2020). The Muslim Turkish couple, cofounder of BioNTech, behind Pfizer vaccine was also applauded in many anti-vax tweets in the post-vaccine period, and they were used as examples to condemn Islamophobia and the fear of immigrants. These tweets were misclassified as anti-vax by the stance detection tool, possibly due to the negative words of condemnation.

The topic of ‘**use of endangered animals**’ talked about the use of horseshoe crab [48] in safety testing and use of squalene, a natural oil found in shark liver [49]. However, only 0.1% of anti-vax tweets are associated with this topic. The topic of ‘**unethical trials**’ contains tweets making accusations that the volunteers are used as ‘lab rats’ and ‘guinea pigs’ for an ‘unsafe vaccine’. A large number of tweets were observed on January 24, 2021, protesting against the use of children in clinical trials, a few days after Pfizer had announced that the trials are fully enrolled [57]. A previous peak on September 7, 2020, is attributed to the news about two historically black American universities urging their students to volunteer for the vaccine trials [58].

## Conspiracies

As given in Table [1](#_bookmark7), we have selected 10 different topics which clearly concern conspiracies. Their evolution over the year-long study period is shown in Figure [4](#_bookmark12) (c, e, g). It is interesting to see that altogether, these conspiracies were discussed in only 6.6% of the total anti-vax tweets during the study period.

The stacked plots in Figure [4](#_bookmark12) use different data ranges for y-axis for clear visualisation. The number of tweets per day for conspiracies are much smaller in comparison to the significant anti-vax topics, such as, ‘side effects’, ‘vaccine mandate’, etc. Moreover, except for the topic of ‘Experimental mRNA/altering DNA’, all conspiracies have mostly lost their momentum towards the end of the study period.

The interest in the topic of **Experimental mRNA/altering DNA** [59], i.e., the conspiracy that mRNA technology can alter human DNA in vaccinated individuals, remained small in pre-vaccine period, and it became more prominent after Pfizer’s announcement of the results of their preliminary trials [34]. The plot shows multiple spikes in the post-vaccine period, but we were not able to identify specific events to correlate. We did notice that such peaks were usually due to a viral meme or tweet with false information, along with a couple of thousand retweets, and this would create a spike due to the overall low volume of tweets for this topic. For our study period, less than 2% of the anti-vax tweets talked about mRNA technology altering DNA. Many fact-checking reports also appeared in the mainstream media, such as, in the BBC, Reuters, etc. Tracing the source of the controversy, one such BBC report [9] talked about a popular YouTube video and in one of their reports, Reuters [13] discussed a preprint from MIT, published on December 13, 2020, on the NIH’s (US National Institute of Health) website about a related controversial study. We have found no significant indication that the MIT study is being discussed within our dataset. The YouTube video, however, was mentioned in a couple of hundred tweets.

The second significant conspiracy featured in our dataset is the conspiracy that COVID-19 vaccines contain **microchips** [60] or tracking devices. A Yahoo/YouGov survey published on May 23, 2020 found that 28% of US adults believed that Bill Gates was plotting to use a mass COVID-19 vaccination campaign to implant microchips in billions of people [61]. We also observed multiple peaks in the ‘microchip’ plot on May 25, 2020. The peak on June 25, 2020 can be attributed to the announcement from the Bill and Melinda Gates Foundation that $106 million funding would be provided to the GAVI vaccine alliance on June 4, 2020. An investigation by the multimedia company ‘The Verge’ [62] suggests that this conspiracy has its origin around the information that Bill Gates and his foundation supported and had interest in projects exploring e-vaccine cards or invisible ‘quantum dots’ containing vaccination history for individuals.

One peak in the microchip plot on July 8, 2020 is also caused by Kanye West’s statement that COVID-19 vaccines contained microchips which were the ‘mark of the beast’ [63]. The microchip theory saw a lot of interest in the beginning of the post-vaccine period, but it lost prominence towards the end of our study period. Moreover, there were many tweets which were pointing out the logical fallacy of the microchip theory by noting the fact that other forms of personal identity and devices could be better means for tracking if there was indeed an agenda to track people. The tallest peak for the microchip plot came on December 8, 2020, on the same day UK started its vaccination program. This peak was due to a made-up viral joke about a vaccine recipient feeling happy that she would never be lost, and the fact that Bill Gates could track her from now on. Shared over 2000 times, it is also not an anti-vax tweet, but rather a satire tweet that was misclassified. Considering the limitation of our stance detection tool, which is not perfect at classifying satire and convoluted expressions, the contribution of the microchip conspiracy to the anti-vax discussion is likely to be even less than what is shown in Figure [4](#_bookmark12)c and Table [1](#_bookmark7).

The very significant peaks in the plot of the topic ‘**fertility**’ [64] in Figure [4](#_bookmark12)c occurred on December 5, 2020 and on December 24, 2020. This anti-vax topic is about the claim that COVID-19 vaccines may cause female/male infertility or sterilisation. The first peak is due to a report that appeared on a German website on December 1, 2020 [65]. This report discussed how Dr. Yeadon, ex-Pfizer executive, had petitioned to stop the COVID-19 vaccine trials for safety concerns, including especially concerns about infertility in vaccinated women [14]. The second peak was mostly discussions regarding an article from a local news outlet of Miami [66], which reported a study about the effects of COVID-19 vaccines on male fertility. While there were no reported results, the last line of the news article was tweeted again and again: "To protect fertility, some men may want to consider freezing their sperm prior to vaccination". These articles created large peaks over Twitter for a couple of days with approximately 5000 and 8000 tweets, but then the discussions about ‘fertility’ died down quickly.

Within this dataset, **Robert F. Kennedy** [67] appeared as the most influential anti-vaxxer. Son of former US Attorney General Robert F. Kennedy and nephew of President John F. Kennedy, Robert Kennedy is actively involved in campaigns against childhood vaccinations. His tweets, including the hashtag #TheDefender and subsequent responses significantly rose in the post-vaccine era. One peak in the topic plot of ‘Robert F. Kennedy’ came on January 23, 2021 because of a viral tweet that blamed the death of former basketball player Hank Aaron (which occurred the day before) on COVID-19 vaccine. The article referenced in the tweet is from Kennedy’s website (childrenshealthdefense.org). The peaks in the plot of ‘Robert F. Kennedy’ usually occurred when articles from Kennedy’s website were shared repeatedly over Twitter.

The conspiracy that COVID-19 vaccines used cells from **aborted babies** also circulated over Twitter, although fewer than 1% of the anti-vax tweets talked about the topic of ‘aborted babies’. The facts, as discussed in [68], are that two cell lines were developed in 1970 and in 1995 from past abortions. These cell lines are permanently established cell cultures, that can propagate repeatedly given fresh medium and space [69]. AstraZeneca and Johnson & Johnson vaccines are developed in one of these cell lines each. The Vatican approved the use of vaccines using these cell lines, as reported by Associated Press on December 22, 2020 [70]. This news was shared in satirical tweets, with quotes around morally acceptable vaccine, and caused a peak in the topic plot on the same day in Figure [4](#_bookmark12)c. March 4, 2021 also witnessed a peak in the plot which can be traced back to the statement from the US Conference of Catholic Bishops, expressing concern over the Johnson & Johnson vaccine [10]. An earlier peak on October 9, 2020, was due to news coverage about Donald Trump receiving treatment for his COVID-19 infection using an anti-viral cocktail that had been tested using the same cell line [11].

The peak in the topic ‘**related to flu vaccine**’ on July 17, 2020 in Figure [4](#_bookmark12)c was due to a viral tweet which commented that “The COVID-19 death rate without a vaccine is lower than the flu death rate with a vaccine". According to WHO’s website [71], at the start of this week, July 13, 2020, the global case fatality rate (number of deaths per confirmed number of COVID-19 cases) was at 4.62%, while the CDC [72] had recorded 28,000 flu deaths out of 29 million illnesses (less than 0.1%) in the previous year of 2019. The second peak in this topic came on January 16, 2021 and was triggered by discussion around reports that 23 people had died in Norway after receiving a COVID-19 vaccine [100] (see details in the main text). The viral tweet claimed 10,000 times increase in the number of flu deaths in Norway during the pandemic year 2020. However, Norwegian Institute of Public Health (NIPH) reported slightly negative excess mortality for Norway in 2020 [73], [74].

Within the topic of ‘**metal/nanoparticles in vaccine**’ [75], we classify the tweets which talked about the presence of metal or lipid nanoparticles in mRNA vaccines. The mRNA vaccine contains lipid nanoparticles, which are organic compounds used to deliver the drug or vaccine to the right place in cells [15]. The term nanoparticle was grossly misrepresented in anti-vax tweets, with many tweets suggesting that such nanoparticles were toxic metal particles, or tiny robots or nanotechnology controlled by 5G cellular towers. The first peak of the topic plot came on December 7, 2020 due to a viral tweet, questioning why Hydroxychloroquine (HCQ) was considered unsafe, but lipid nanoparticles were considered safe. Interestingly, this peak came right after the publication of the Reuters’ fact-check report [15]. The controversy regarding Hydroxychloroquine is discussed in detail later in the topic ‘HCQ/Ivermectin’. There were multiple scientific studies that resulted in many countries suspending or stopping its use as a COVID-19 treatment [35]. Another peak in this plot on January 29, 2021 is also attributed to tweets which misrepresented a 2018 peer-reviewed publication about the use of lipid nanoparticles for brain targeting [76].

The origin of a conspiracy that COVID-19 vaccines can **make a person gay** in our dataset is mostly attributed to two clerics. An Israeli newspaper [77] reported in an article on January 11, 2021, about a rabbi instructing his online followers that COVID-19 vaccines can make them homosexual. The article from the newspaper was in fact refuting the claim, and the news was widely shared worldwide, mostly by tabloid press. We saw a peak in the topic plot on January 17, 2020, (in Figure [3](#_bookmark12) (d] sharing the link of the story from a queer website [78]. On February 10, 2021, an Israeli newspaper, The Jerusalem Post, shared the news of an Iranian cleric with a similar claim [79]. This news article was widely shared by mainstream media globally, again mostly by tabloids, and caused a peak in the ‘vaccine can make you gay’ topic plot shortly after, on February 15, 2021. These news articles seem to be condemning homophobia rather than advancing a conspiracy against COVID-19 vaccine. However, the tweets sharing the news articles were labelled ’anti-vax’ by our annotators due to the negative message about the vaccine. According to the news articles, this conspiracy was mostly propagated over the private online groups of these clerics.

Only 0.1% of the anti-vax tweets have been separately assigned the topics of **QAnon** and **eugenics**. The proponents of QAnon, a political conspiracy theory [80], also supported multiple anti-vax conspiracies including vaccine is making people gay, vaccine is a bioweapon, COVID-19 tests contain vaccine, Bill Gates has a depopulation agenda through vaccination, etc. Our stance detection tool also misclassified many tweets which contained satirical condemnation of the talking points put forward by QAnon.

Eugenics is an idea and set of practices that concerns attempts to improve the genetic quality of the human population by selective breeding. Coined by Francis Galton in 1883, eugenics got considerable support from academia and general population in the late 19th and early 20th centuries, as described in [81]. For the context of COVID-19 vaccine, we found tweets which claimed that COVID-19 vaccines were a eugenics agenda driven by Bill Gates, Anthony Fauci, Oxford University, etc.

# Lesser Discussed Common Topics

## COVID-19 responses

The sub-topic ‘**misc. COVID responses**’ include a broad range of initiatives that authorities have taken to inform the public about vaccine safety, controlling the spread of coronavirus, addressing the financial implications of lockdowns, postponing exams, and sporting events, etc. The anti-vax tweets mostly contained negative news about the vaccine and were critical of the response measures. The pro-vax tweets were mostly focused on updates about COVID relief measures, vaccine access and distribution, etc.

Vaccine access, patents and IP issues, and priority order are the main discussion points in ‘**access to vaccine/priority order**’. The proposed approach of letting COVID-19 disappear naturally by exposing a critical mass of the population to the pathogen is discussed within the topic of ‘**herd immunity through infection**’. The pro-vax tweets were usually critical of this approach, while the anti-vax tweets were raising the issue that, assuming infection from COVID-19 could not provide immunity, then how could a vaccine provide protection. Sweden was praised by the anti-vax tweets, and lockdowns were deplored.

## Vaccine Candidates

We have discussed Pfizer/Moderna in the main text. The anti-vax tweets about ‘**AstraZeneca**’ mostly discussed the adverse effects reported during clinical trials and public distribution. Use of the AstraZeneca vaccine was suspended across many countries on March 11, 2021, some temporarily suspended the vaccine and for certain age groups [82]. A high volume of anti-vax tweets related to AstraZeneca was observed on March 15, 2021, followed by a high peak in pro-vax tweets a day later. These pro-vax tweets, in general, supported the AstraZeneca vaccine by referring to the blood clotting potential of other drugs, such as, contraceptive pills or pointing out the small rate of blood clot incidents after vaccination.

**Johnson & Johnson** (J&J) vaccine is the least discussed vaccine in our dataset. We remark that the relevant tweets are even less than the number given in Table [1](#_bookmark7), due to misclassification of tweets referring to Boris Johnson into this topic. In this topic, the pro-vax tweets mostly discussed the development updates, but many anti-vax tweets referred to the J&J’s baby powder causing cancer lawsuit [83]. Similar to discussions on other COVID-19 vaccines, the issues of safety, money making potential, etc. were also discussed in the context of J&J’s vaccine.

## People

The discussion about **Dr. Anthony Fauci** and CDC was clearly polarised between the anti-vax and pro-vax groups. The former was critical and considered him to be co-conspiring with Bill Gates for probable monetary interests, whereas the latter expressed their trust over the decisions by Dr. Fauci and the CDC.

We started hydrating the Lamsal’s dataset [27] after Twitter banned **Trump**, and it was not possible to access his archived tweets and all subsequent retweets. He was discussed in only 3.4% of anti-vax and 4.5% of pro-vax tweets, but we suspect that these numbers do not show the real picture, and some relevant tweets might not have retrieved. The plot peak on September 16, 2020 can be traced to Trump’s statement that contradicted the statements of the CDC, in relation to the timeline for vaccine development [84].

## Countries

While many countries were mentioned in the tweet dataset, **China** and **Russia** came out as the two most prominent topics of discussion, especially for anti-vax tweets. Here, we comment on both countries/topics. Since SARS-CoV-2 was first detected in

Wuhan, China, we found many anti-vax tweets referring to COVID-19 as the ‘China-virus’ or ‘CCP-19’ (Chinese Communist Party). There was also criticism of the fast-paced vaccine development in China. The pro-vax tweets discussed Chinese vaccine development, updates about distribution, vaccine IP issues, etc. Russia was marginally discussed in our tweet dataset. The anti-vax tweets were critical of the Russian vaccine, especially regarding the skipping of the phase 3 trials, and pro-vax tweets were talking about vaccine development updates.

## COVID-19 Treatments

Within this topic, the most significantly discussed treatment is **Hydroxychloroquine (HCQ)**. Initially trialed as a possible cure [85], HCQ later became a controversial topic. There were multiple scientific studies providing evidence to governments and policymakers that resulted in many of them stopping its use as a COVID-19 treatment [35]. However, a number of anti-vax tweets suggested that HCQ was banned only to give EUA to COVID-19 vaccines. The discussion focused on the fact that HCQ and **Ivermectin** are cheaper drugs and would not bring comparable profits as COVID-19 vaccines to pharmaceutical companies. Pro-vax tweets on the other hand talked about HCQ and other therapeutics in a positive way and discussed their updates along with that of vaccines. **BCG** vaccine and **antibodies/plasma from COVID survivors** were trialed initially in the pandemic, and both remained uncontroversial in our dataset.

## Marginally Discussed Common Topics

Within the topic ‘**race**’, the anti-vax tweets were mostly emphasising the historic discrimination against African Americans. The stories of the Tuskegee study [40] and Henrietta Lacks [41] were mentioned in multiple tweets and a statement from African American leader Louis Farrakhan, where he asked black people to get a COVID-19 vaccine only if it was made by black scientists, was retweeted a number of times. The pro-vax tweets, on the other hand, discussed the Black Lives Matter movement in the context of COVID-19 and talked about the disproportionate impact of COVID-19 on coloured communities in the US.

Under the topic of ‘**social media/mainstream media**’, both anti-vax and pro-vax communities criticised the role of media, especially social media. The pro-vax tweets were critical of the propagation of unverified information over social media platforms, and anti-vax tweets were complaining about lack of freedom of speech over these forums.

The remaining topics given in Table [1](#_bookmark7), such as, ‘**Spanish flu**’ and ‘**elections**’ were marginally discussed. The topic ‘Spanish flu’ concerns the discussion about the 1918 influenza pandemic, and the topic of ‘elections’ covered the tweets discussing US presidential elections 2020 and Indian state elections and by-elections for Lok Sabha in October-November 2020.

# Lesser Discussed Pro-vax Topics

The topic ‘**debunking anti-vax conspiracies**’ also showed multiple peaks during December 15-21, 2020, and is due to a viral meme about vaccine ingredients. The topic ‘**other viral infections**’ contains tweets that refer to diseases such as polio and smallpox, which were successfully contained by vaccination efforts. The topic of ‘**vaccine mandate (pro-vax)**’ covers tweets that discuss support for the idea of mandatory vaccines to curb the spread of coronavirus. Within the topic of ‘**climate change**’, the discussion is about tackling global challenges, i.e., COVID-19, climate change, empowering women, etc. ‘**Globalisation**’ tweets talked about global efforts to successfully develop and distribute COVID-19 vaccines and ‘**major events**’ was mostly focused on the postponement of 2020 Tokyo Olympics due to the COVID-19 pandemic.

# Interesting themes

While working on topic classification, we came across some themes, such as the use of swear words or discussions about trust, which cannot be categorised as discussion topics. Nonetheless, comparing such themes across the two classes of anti-vax and pro-vax tweets can give us more insight about the groups’ collective attitude or characteristics. A detailed linguistic comparison is outside the scope of the current paper. We have selected 6 *secondary* themes:

1. **Trust** We tracked the use of keyword ‘trust’.
2. **Voting ID and mail-in voting** Near the US elections, we observed tweets in the anti-vax group, arguing why Democrats were against voting IDs and in favour of mail-in voting but would not want the same for the vaccine.
3. **Deep state** We tracked the use of keywords, such as, ‘globalist’, ‘elite’, ‘deep state’, ‘new world order’.
4. **Governments** Here, we tracked the names of government officials and agencies used in tweets.

0.1

Fraction of Tweets

**Trust**

4

10-3 **Voting ID & mail-in voting**

pro-vax anti-vax

3

0.05 2

1

0

0.6

Fraction of Tweets

**Deep state**

0

0.6

**Governments**

0.4 0.4

0.2 0.2

0

0.1

Fraction of Tweets

**WHO**

0

0.2

**Swear words**

0.15

0.05 0.1

0.05

0 0

10 20 30 40 50

Weeks

10 20 30 40 50

Weeks

**Figure 6.** Interesting themes to compare in anti-vax and pro-vax classes.

1. **WHO** We tracked the mentions and references to the World Health Organisation.
2. **Swear words** Under this topic, we tracked the use of swear words and/or derogatory words in the tweets.

Figure [6](#_bookmark36) shows the plots of pro-vax and anti-vax tweets for each of the secondary theme. Because of class imbalance between the two groups, we plot the fraction of tweets per week, where the tweets in a week are divided by the total tweets in the anti-vax/pro-vax group for the week. Looking at Figure [6](#_bookmark36), it is clear that ‘deep state’ is discussed solely in the anti-vax group. The very large peak in anti-vax plot for ‘deep state’ at week 4, came in context of Bill Gates (see main text).

From the Figure [6](#_bookmark36), ‘voting ID and mail-in voting’ also looks like an overwhelmingly anti-vax theme. The first pro-vax peak in the chart came in week 21 and is due to tweets which accuse US Democrats of dragging the debate on COVID relief package in the US House of Representatives to also consider no-ID voting. The first anti-vax peak which came right after the pro-vax peak, in week 24, is due to tweets that report a robocall scam with misinformation to deter mail-in voting [86] These tweets are misclassified as anti-vax. The second peak in week 39, are true anti-vax tweets which came in the midst of Trump’s lawsuits against alleged election fraud, with the tweets claiming that COVID-19 was an excuse for mail-in voting and also claiming that vaccines should also be mailed to US residents. Similar discussion resulted in the peak for pro-vax plot in week 51, however, the mail-in vaccine idea should be classified as anti-vax and we consider it as another misclassification by the stance detection tool. The last anti-vax peak in week 52 consisted of tweets that accused US Democrats for pushing for vaccine ID to estimate *zombie voters* for next election. These tweets are possibly supporting multiple conspiracies: 1- that zombie voters, i.e., voters who are not alive [87], vote for Democrats [88], and 2- people who take COVID-19 vaccine will die and become zombies.

Names of government officials and WHO are mentioned in almost similar proportions in both anti-vax and pro-vax groups. However, the use of the word ‘trust’ and use of swear words were more prominent in the anti-vax group compared to the pro-vax group. The very large peak in pro-vax plot for ‘swear words’ on week 14 is due to a tweet (and its retweets) *damning* the ongoing situation in which people would not be able to afford the COVID-19 vaccine. Even with this peak, the pro-vax group recorded 2.7% of tweets using one or more swear words, compared to 5.4% tweets in the anti-vax group. Our findings suggest that anti-vax tweets are more emotional than pro-vax tweets, but further investigation, such as [89], is

required to understand the salient attributes of the user groups.

# University of Melbourne Dataset


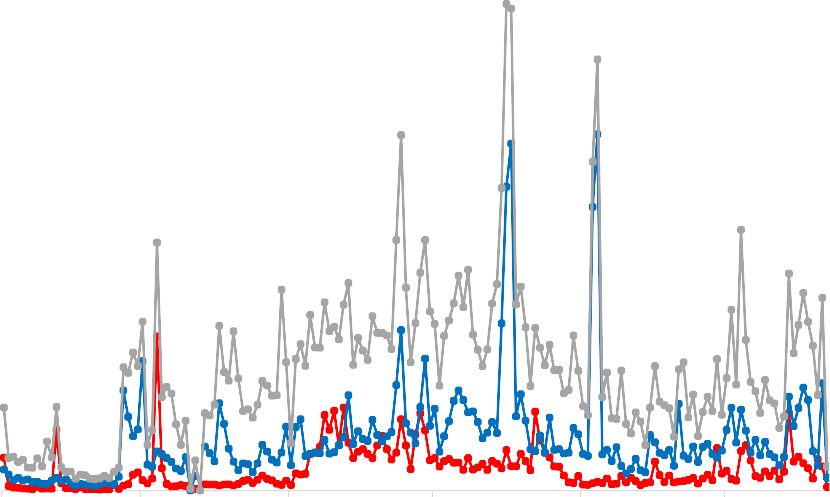


100000

90000

80000

70000

60000

50000

40000

30000

20000

10000

0

31-Jan-20

29-Feb-20

31-Mar-20

30-Apr-20

31-May-20

30-Jun-20

Favour Against Total relevant

**Figure 7.** Stance detection results for Twitter dataset collected at University of Melbourne (UoM) from January 31, 2020 to July 20, 2020. Pro-vax tweets are 2.5 times more than anti-vax tweets for this dataset.

As discussed in the main text, we were not able to hydrate all tweet IDs from Lamsal’s dataset [27]. In order to ascertain that the loss in hydration does not affect our observations and conclusions, We have also used another independently collected data set with COVID-19 related keywords^[[1]](#footnote-1)^, some keywords are similar to [27]. This data set is collected at University of Melbourne (UoM) using their RAPID platform [90] from February-July 2020, which saved all fields of the tweet object. We have further filtered this dataset for English language tweets and with vaccine related keywords (vaccine, vaccination, vax, vac, jab, and shot). The labelled training sets developed for Lamsal’s dataset were also used to train the GPT stance detection tool for stance prediction of UoM tweets. The results are shown in Figure [7](#_bookmark37). This dataset also revealed 2.5 times more pro-vax tweets than anti-vax for the study period. Moreover, similar peaks are observed in Figure [7](#_bookmark37) for the same period, as in Lamsal’s dataset. For example, the peak related to Bill Gates on April 9, 2020 is present in UoM dataset as well.

Figure [8](#_bookmark37) shows the topic classification for UoM dataset, similar to the one presented in the main text, using the topic modeling tool GS-DMM [19]. These results are similar to the results presented in the main text for Lamsal’s dataset. Vaccine development is the most significant pro-vax topic and vaccine mandate and side effects are among the top anti-vax topics. However, we observed that the topic ‘Trump’ is ranked higher in this dataset which is expected as we could not retrieve Trump’s tweets and subsequent retweets for Lamsal’s dataset. Another interesting difference is the top anti-vax topic ‘Bill Gates’, which ranked 8^th^ among the anti-vax topics discussed in the main text. In our opinion, this difference is because of the different time periods of tweet collection. Bill Gates was the main focus of anti-vax tweets in the beginning of the pandemic but then this interest faded away. As discussed in the main text, anti-vax content has a broader range of discussion points, whereas pro-vax posts are largely about vaccine development updates.

Figure [9](#_bookmark37) shows the composition of anti-vax tweets in terms of tweets which only contained falsehoods versus tweets which touched upon some genuine issue. Similar to the main text, where the contrast is shown for Lamsal’s dataset, Figure [9](#_bookmark37) shows that anti-vax tweets have a significant dose of misinformation and falsehoods.


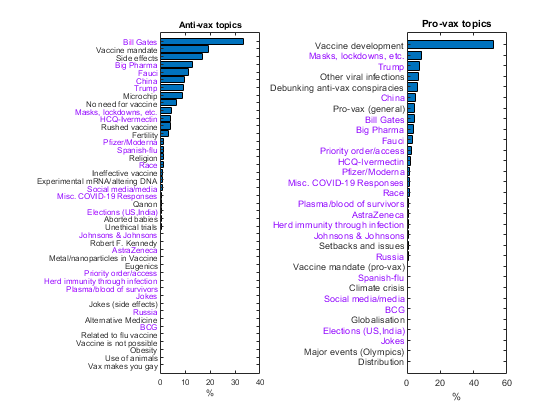


**Figure 8.** Anti-vax and pro-vax topics for UoM data. Topics are arranged according to the fraction of tweets containing them. Vaccine development is the most significant pro-vax topic and vaccine mandate and side effect are among the top anti-vax topics as in Lamsal’s data set [27]. The topic Trump ranked higher in UoM dataset which is expected because Trump’s tweets and subsequent retweets were already deleted when we hydrated Lamsal’s dataset. The interesting difference is the top anti-vax topic of Bill Gates which is due to the period when UoM tweets were collected. Anti-vax tweets were focused on Bill Gates during the initial months of the pandemic.


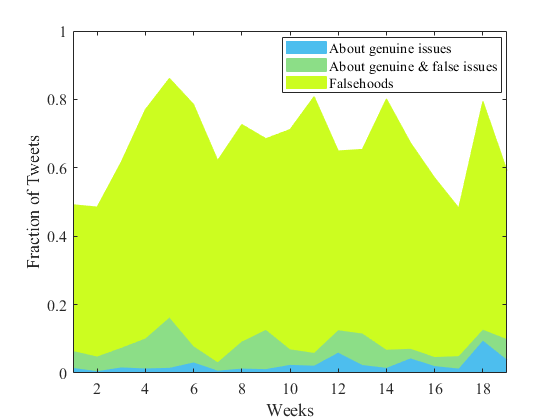


**Figure 9.** Falsehood and misinformation versus posts referring to genuine issues in UoM dataset. As in Lamsal’s dataset, the anti-vax content is largely consistent of falsehoods and misinformation.

# Annotation Guidelines

The following guidelines have been considered by all annotators while labeling the training and test sets.

## Anti-vax

1. Harmful side effects of vaccines
2. Post-vaccine deaths
3. Vaccine as a very lucrative business proposition or Covid is engineered so vaccine can be justified.
4. Famous people such as Bill Gates and Scott Morison are claimed to have shares in big pharma behind vaccine manufacturing and distribution. Big pharma profits
5. Key themes: 5G antennas in body, microchip, autism, changing DNA, experimental, devils mark, depopulation agenda, getting tracked, aborted babies, unethical, threat of mandatory vaccination.
6. Doubts about having vaccine, example: “I’m curious. Do you plan on getting the ??COVID19?? vaccine?”
7. Vaccine can make you more ill.
8. Ridiculing vaccine with jokes and memes or sarcasm
9. Against vaccination process in general
10. discredit Hydroxychloroquine in order to build a case for vaccine.
11. questions like ‘will you take a vaccine?”
12. Suspicious media hype about vaccine
13. No need for vaccine because there is some natural remedy/prayer etc. Or covid is similar to cold and there is no vaccine for cold.
14. Plandemic, Gate’s vaccine, new world order
15. Experimental vaccine, gene therapy, changing DNA.
16. Emphasis on speed of production, such as operation wrap speed, fuels skepticism
17. Bad consequences of vaccination in general
18. Human guinea pigs for vaccine tests
19. Don’t count on vaccine for Covid-free world.

## Pro-vax

1. Vaccine development updates
2. Information and discussion about vaccine
3. Funding for vaccine, Philanthropic funding including Bill and Melinda Gates Foundation’s funding (which is used as an anti-vax argument too)
4. Responses against anti-vax posts or debunking anti-vax conspiracy theories.
5. Free/easy access to vaccine, open patents
6. Covid19 is more dangerous because it has no vaccine.

Example: “This is NOT “just the flu”: ??We have lost more lives to #COVID19 than during the entire past three flu seasons COMBINED ??You are 100 times more likely to be hospitalized ??Unlike the flu, #COVID19 has no vaccine and no proven therapeutics <https://t.co/rjSjyVaeXL>”

1. Praising those (people, countries, companies) who are working to develop the vaccine.
2. Herd immunity without vaccine is a bad idea.
3. In favour of vaccination and inoculation idea in general
4. Example: Sixty per cent of Canadians polled say that a coronavirus vaccine should be mandatory. Do you agree?
5. Media response during BLM protests forgetting Covid and vaccines.

## Neutral

1. Irrelevant
2. Neutral
3. Negative but true facts, example: “I don’t know why people keep saying a vaccine for Covid-19 is a year away. It could be, or we could never find one. When it was discovered that HIV caused AIDS in 1984, government researchers told us that we’d have a vaccine in 2 years. 36 yrs later and, we’re *still* searching…”
4. Talk about issues in distribution, racial inequality, incompetence of different government agencies, etc.
5. Unclear, example: “Our Herbalists should delve into research on finding vaccine for #COVID19 rather than so much concentration on production and marketing of ‘manpower’ drugs.” Although the tweet seems pro-vaccine but traditional herbalist is not trained to develop vaccine by following standard scientific processes.
6. Politics around vaccine, such as, Trump/Modi developed vaccine, Trudeau and Morrison have bungled up the distribution, democrats are taking the credit, etc.
7. Use of prisoners, politicians, etc., for vaccine trials.
8. There is no vaccine for racism, authoritarian rule, etc.
9. Vaccine’s business prospects
10. Jesus is vaccine or Ramadan is vaccine.
11. Caution against or news about fake vaccines
12. Fear about not finding an effective vaccine ever.
13. Irrational statements, specially from President Trump about vaccine, such as those who developed vaccine for AIDS will develop one for Covid19. There is no vaccine for AIDS.
14. Promising treatments instead of vaccine.
15. Speeding up vaccine in time for election.
16. Jokes about fake vaccines

1. #coronavirus, covid19aus, pandemic, virus, christmas, #2019ncov, wuflu, #coronavirusaustralia, christmasisland, christmas island, #covid19aus, coron- avirusaustralia, coronavirus, corona, covid-19 [↑](#footnote-ref-1)
